# Mutation of the *PTCH1* gene predicts recurrence of breast cancer

**DOI:** 10.1038/s41598-019-52617-4

**Published:** 2019-11-08

**Authors:** Chih-Yang Wang, Yung-Chieh Chang, Yao-Lung Kuo, Kuo-Ting Lee, Pai-Sheng Chen, Chun Hei Antonio Cheung, Chih-Peng Chang, Nam Nhut Phan, Meng-Ru Shen, Hui-Ping Hsu

**Affiliations:** 1 Institute of Basic Medical Sciences, College of Medicine, National Cheng Kung University, Tainan, Taiwan; 2 Department of Pharmacology, College of Medicine, National Cheng Kung University, Tainan, Taiwan; 3 Department of Surgery, National Cheng Kung University Hospital, College of Medicine, National Cheng Kung University, Tainan, Taiwan; 4 Department of Medical Laboratory Science and Biotechnology, College of Medicine, National Cheng Kung University, Tainan, Taiwan; 5 Department of Microbiology and Immunology, College of Medicine, National Cheng Kung University, Tainan, Taiwan; 6 NTT Institute of Hi-Technology, Nguyen Tat Thanh University, Ho Chi Minh City, Vietnam; 7 Department of Obstetrics and Gynecology, National Cheng Kung University Hospital, College of Medicine, National Cheng Kung University, Tainan, Taiwan

**Keywords:** Breast cancer, Molecular medicine, Breast cancer

## Abstract

Breast cancer is the most common cancer in women, and some patients develop recurrence after standard therapy. Effective predictors are urgently needed to detect recurrence earlier. The activation of Hedgehog signaling in breast cancer is correlated with poor prognosis. *PTCH1* is an essential membrane receptor of Hedgehog. However, there are few reports about mutations in Hedgehog genes in breast cancer. We conducted a comprehensive study via an experimental and bioinformatics approach to detect mutated genes in breast cancer. Twenty-two breast cancer patients who developed recurrence within 24 months postoperatively were enrolled with 22 control cancer patients. Targeted deep sequencing was performed to assess the mutations among individuals with breast cancer using a panel of 143 cancer-associated genes. Bioinformatics and public databases were used to predict the protein functions of the mutated genes. Mutations were identified in 44 breast cancer specimens, and the most frequently mutated genes were *BRCA2, APC, ATM, BRCA1, NF1, TET2, TSC1, TSC2, NOTCH1, MSH2, PTCH1, TP53, PIK3CA, FBXW7*, and *RB1*. Mutation of these genes was correlated with protein phosphorylation and autophosphorylation, such as peptidyl-tyrosine and protein kinase C phosphorylation. Among these highly mutated genes, mutations of *PTCH1* were associated with poor prognosis and increased recurrence of breast cancer, especially mutations in exons 22 and 23. The public sequencing data from the COSMIC database were exploited to predict the functions of the mutations. Our findings suggest that mutation of *PTCH1* is correlated with early recurrence of breast cancer patients and will become a powerful predictor for recurrence of breast cancer.

## Introduction

According to the Cancer Statistics Report in 2018, breast cancer constitutes 30% of new cancer cases diagnosed in American women^1^. The Centers for Disease Control and Prevention estimated that cancer will surpass heart disease to become the primary source of U.S. mortality by 2020. In 2014, the Taiwan Cancer Registry reported a breast cancer incidence of 11,769 cases per 100,000 females in Taiwanese women^2^. Although many therapeutics have been applied to treat breast cancer patients, some patients developed recurrence rapidly after standard treatment. Besides traditional classification, no effective predictors can be used to detect recurrence in chemoresistant breast cancer patients. There is also a lack of suitable treatment for these patients.

Cancer is a complex disease arising from the sequential accumulation of somatic mutations leading to the transformation of normal cells to cancerous cells. Detection of novel somatic mutations is important for examining the mechanism of carcinogenesis and to seek proper treatments^3,4^. Because of cancer heterogeneity, identifying somatic mutations from tumor samples is more difficult than detecting germline mutations using purified peripheral blood. Intercellular and intratumor genetic heterogeneity are the main sources of complications for identifying low-frequency mutations. However, somatic mutations of cancer samples are also important in clinical applications, including initial diagnosis, long-term monitoring, and relapse identification. All of these are essential components of cancer treatment. Detection of low-frequency mutations is also desirable in newly developed applications^5–7^.

Cancer is a genetic disease caused by both acquired and inherited mutations^8^. Incorporation of genome sequencing of cancer samples and online information from large data sets may accelerate the process of detecting new predictors. Solid evidence has been provided from cancer samples and sufficient references from online information^9,10^. It is important to integrate genetic alterations, cancer phenotypes and clinical outcomes of patients^11,12^. The present study involved two main steps. First, we analyzed the variations of 143 genes from 44 samples of breast cancer patients in Taiwan and defined several highly mutated genes. Furthermore, we compared the public datasets of these highly mutated genes from TCGA (The Cancer Genome Atlas)^13^, METABRIC (Molecular Taxonomy of Breast Cancer International Consortium)^14^, COSMIC (Catalogue of Somatic Mutations in Cancer)^15^, and NCBI GEO (National Center for Biotechnology Information, Gene Expression Omnibus)^16^. The findings were correlated with clinical outcomes and recurrence-free survival of breast cancer patients.

## Results

### Mutation burden versus the fraction of the genome altered in breast cancer patients

**Figure 1:**
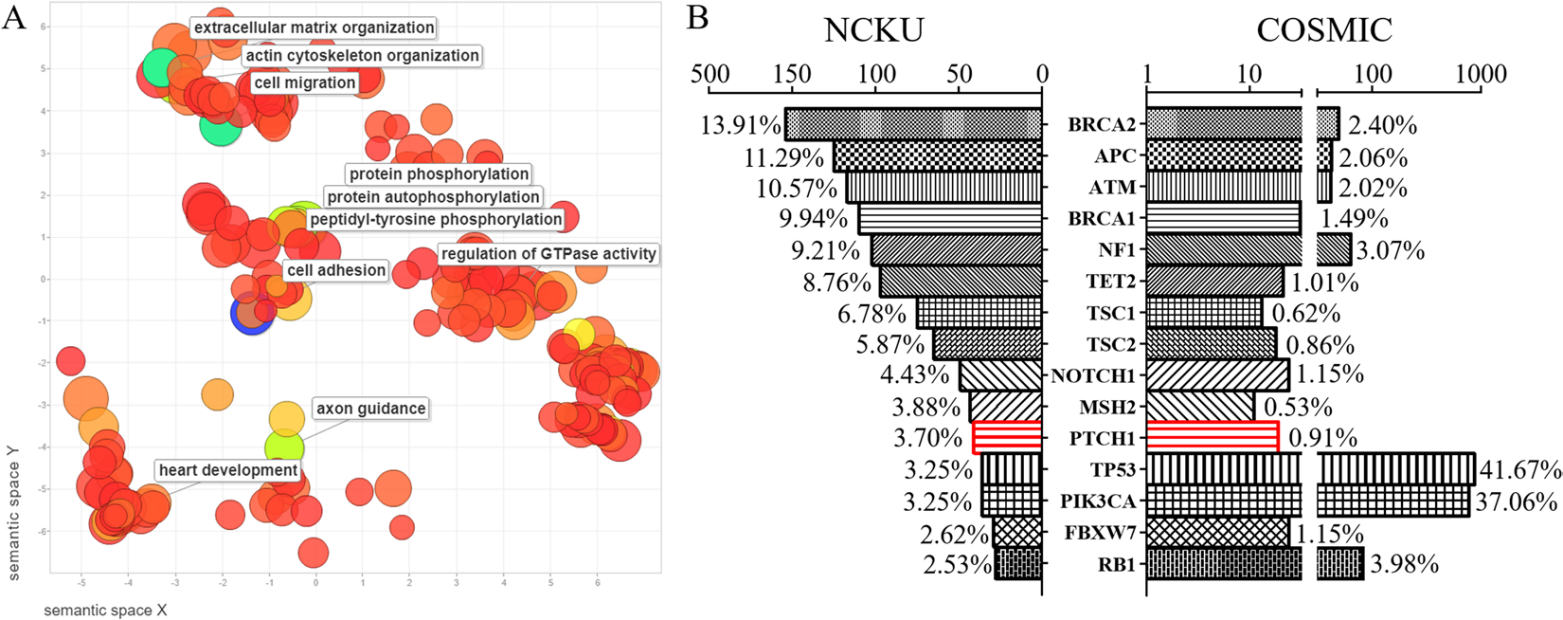
Overview of frequently mutated genes in the present study and comparison with the COSMIC database of breast cancer. (**A**) The significantly enriched Gene Ontology (GO) terms were calculated by CLC Genomics Workbench according to the mutated genes from sequencing data, and the graph was plotted with the REViGO package for the top ten significant GO terms. Each of the GO terms represents a node in the graph. The scatterplot showing functional clusters according to biological processes and the bubble color refer to the *p*-values from the GO result, whereas the bubble size indicates the frequency of the biology process from the GO database. The larger bubbles have more general terms, and these bubbles were highly correlated with protein phosphorylation, such as peptidyl-tyrosine phosphorylation, protein phosphorylation, and protein autophosphorylation. (**B**) Mutation prevalence in our cohort and comparison with the COSMIC database. The X-axis is the total number of mutations in the particular gene. The percentage close to the bar is the number of mutations in the particular gene divided by sum of all mutations in these 15 genes.

A total of 22 patients with cancer recurrence within 24 months postoperatively were collected (recurrence group). Twenty-two matched patients without cancer recurrence were selected as the control group. Breast cancer tissue was analyzed by targeted deep sequencing for mutations in 143 cancer-related genes (Supplementary Table S1). Patient characteristics are shown in Table 1. The patients with cancer recurrence had a larger tumor size than the matched controls (P = 0.044, Table 1). Age, operative method, TNM staging (tumor-node-metastasis), and intrinsic subtypes were similar between recurrence and matched control groups. CLC Genomics Workbench and Gene Ontology (GO) were used to analyze our sequencing data of 44 patients. Enrichment of GO terms among the mutated genes was clustered with top ten of the significant GO terms in the REViGO algorithm (Reduce + Visualize Gene Ontology, Fig. 1A and Table 2). GO terms from the categories “biological processes”, “cellular components” and “molecular function” with an adjusted *p*-value of <0.05 were considered overrepresentative in a subset of analyzed genes. These highly mutated genes had a high correlation with protein phosphorylation, such as peptidyl-tyrosine phosphorylation, protein phosphorylation and autophosphorylation (Fig. 1A). The highly mutated genes also correlated with cell adhesion, cell migration, extracellular matrix organization, axon guidance, actin cytoskeleton organization, heart development, and the regulation of small GTPase-mediated signal transduction (Table 2). Supplementary Table S2 provides all detailed information about the correlation between the novel GO terms and mutation in our breast cancer patients.

**Table 1:** Characteristics of patients with breast cancer (n = 44). Comparison between recurrence and control patients was done.

Characteristic	Recurrence^*^	Control^**^	*p*-value
Patients, n (%)	22 (50%)	22 (50%)	
Age at surgery (years)^***^	47 (29–63)	50 (35–75)	0.289
Tumor size (cm)^***^	3.3 (1.4–8.0)	2.6 (1.5–5.5)	0.044
Operation methods			0.203
Partial mastectomy and sentinel lymph node biopsy	1 (33%)	2 (67%)	
Partial mastectomy and axillary lymph node dissection	1 (100%)	0	
Total mastectomy and sentinel lymph node biopsy	2 (100%)	0	
Modified radical mastectomy	18 (47%)	20 (53%)	
Tumor-infiltrating lymphocytes			
Low			
High			
Histology grade			>0.999
Nuclear grade II	4 (44%)	5 (56%)	
Nuclear grade III	18 (51%)	17 (49%)	
Extensive intraductal components	10 (67%)	5 (33%)	0.203
Lymphatic tumor emboli	13 (52%)	12 (48%)	>0.999
Extranodal extension	6 (67%)	3 (33%)	0.460
Tumor stage			0.493
T1	1 (33%)	2 (67%)	
T2	18 (49%)	19 (51%)	
T3	3 (75%)	1 (25%)	
Nodal stage			0.103
N0	9 (47%)	10 (53%)	
N1	6 (46%)	7 (54%)	
N2	3 (38%)	5 (62%)	
N3	4 (100%)	0	
AJCC TNM stage			0.168
Stage IA	1 (33%)	2 (9%)	
Stage IIA	7 (47%)	8 (53%)	
Stage IIB	7 (50%)	7 (50%)	
Stage IIIA	3 (37%)	5 (63%)	
Stage IIIC	4 (100%)	0	
Intrinsic subtypes			0.716
Luminal A	3 (37%)	5 (63%)	
Luminal B without Her-2/Neu overexpression	3 (50%)	3 (50%)	
Luminal B with Her-2/Neu overexpression	6 (67%)	3 (33%)	
Her-2/Neu overexpression	3 (37%)	5 (63%)	
Triple-negative breast cancer	7 (54%)	6 (46%)	

AJCC TNM stage, American Joint Committee on Cancer tumor-node-metastases (TNM) staging system, 7^th^ ed.

^*^Cancer recurrence within 24 months postoperatively. ^**^No recurrence after 24 months postoperatively. ^***^Values are expressed as median (range).

**Table 2:** Predicting Gene Ontology (GO) biological process according to the mutation data from 44 sequenced breast cancer patients.

GO term	Description	*p*-values
GO:0007155	cell adhesion	1.53E-14
GO:0016477	cell migration	8.63E-11
GO:0030198	extracellular matrix organization	1.01E-10
GO:0018108	peptidyl-tyrosine phosphorylation	1.21E-08
GO:0006468	protein phosphorylation	2.38E-08
GO:0007411	axon guidance	3.83E-08
GO:0030036	actin cytoskeleton organization	3.99E-08
GO:0046777	protein autophosphorylation	5.64E-08
GO:0007507	heart development	6.76E-07
GO:0051056	regulation of small GTPase mediated signal transduction	6.99E-07
GO:0048010	vascular endothelial growth factor receptor signaling pathway	7.79E-07
GO:2000463	positive regulation of excitatory postsynaptic potential	2.34E-06
GO:0009968	negative regulation of signal transduction	2.69E-06
GO:0071300	cellular response to retinoic acid	4.30E-06
GO:0007165	signal transduction	8.22E-06
GO:0043410	positive regulation of MAPK cascade	1.21E-05
GO:0007399	nervous system development	1.34E-05
GO:0010595	positive regulation of endothelial cell migration	1.76E-05
GO:0006024	glycosaminoglycan biosynthetic process	1.98E-05
GO:0060997	dendritic spine morphogenesis	2.64E-05

Enrichment of functionally related GO terms and clustering of significant GO terms was analyzed by CLC Genomics Workbench. The first 20 GO terms are listed.

**Figure 2:**
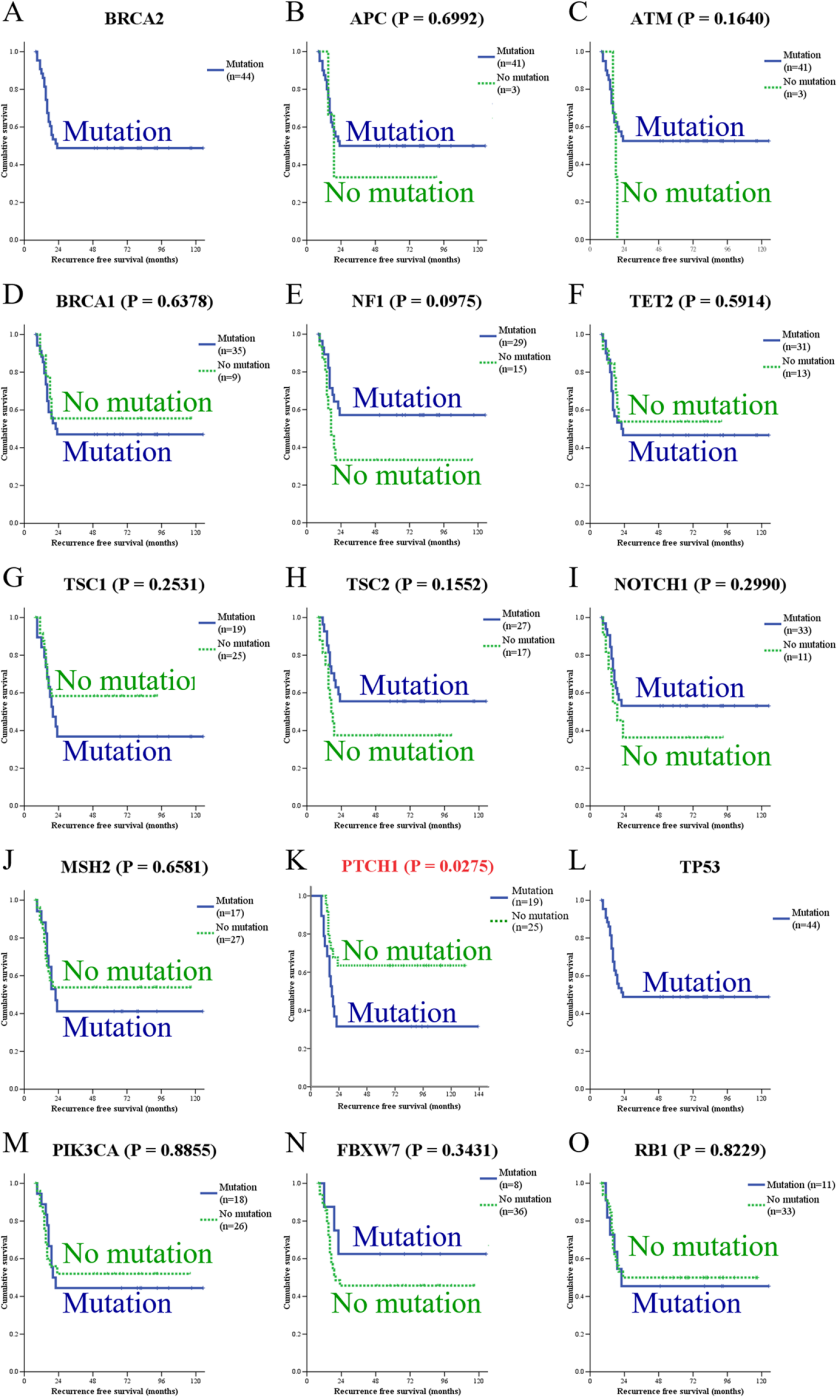
Kaplan-Meier analysis of recurrence-free survival based on the mutation status of each gene. Mutation of *PTCH1* was correlated with poor prognosis.

Selected alterations that appeared in target genes were compared with those in the COSMIC database (containing results from TCGA, METABRIC, and NCBI GEO, Fig. 1B). The results from TCGA was compared separately and listed in Supplementary Fig. S1. Previous studies reported that *BRCA2*, *APC*, *TP53*, and *PIK3CA* were highly mutated genes in breast cancer patients, and these genes were served as positive controls for our analysis^13,14^. Remarkably, we found a high mutation rate of *PTCH1* in Taiwanese breast cancer patients (3.70% in our series vs. 0.91% in COSMIC, Fig. 1B, or 1.39% in TCGA, Supplementary Fig. S1). Among the 15 most often mutated genes, some mutated genes were also correlated with good prognosis, such as *APC*, *ATM*, *NF1*, *TSC2*, *NOTCH1*, and *FBXW7*. Others were correlated with poor prognosis, such as *BRCA1*, *TET2*, *TSC1*, *MSH2*, *PTCH1*, and *PIK3CA*. Mutation of *PTCH1* predicted poor recurrence-free survival with statistical significance (P = 0.0275, Fig. 2). The incidence of *PTCH1* mutation was higher in patients with recurrence compared with nonrecurrence control (68% vs. 34%, Table 3). Since only a few studies have focused on this gene in breast cancer, we selected *PTCH1* for further analyses.

**Table 3:** Correlation of genetic mutations with the two group of patients (n = 44).

Characteristic	Recurrence^*^	Control^**^	*p*-value
Patient, n (%)	22 (50%)	22 (50%)	
*BRCA2* mutation	22 (50%)	22 (50%)	***
*APC* mutation	20 (49%)	21 (51%)	>0.999
*ATM* mutation	19 (46%)	22 (53%)	>0.999
*BRCA1* mutation	18 (51%)	17 (49%)	>0.999
*NF1* mutation	12 (41%)	17 (59%)	0.203
*TET2* mutation	16 (52%)	15 (48%)	>0.999
*TSC1* mutation	12 (63%)	7 (37%)	0.223
*TSC2* mutation	12 (44%)	15 (56%)	0.537
*NOTCH1* mutation	15 (45%)	18 (55%)	0.488
*MSH2* mutation	10 (59%)	7 (41%)	0.537
*PTCH1* mutation	13 (68%)	6 (34%)	0.067
*TP53* mutation	22 (50%)	22 (50%)	***
*PIK3CA* mutation	10 (56%)	8 (44%)	0.760
*FBXW7* mutation	3 (37%)	5 (63%)	0.698
*RB1* mutation	6 (55%)	5 (45%)	>0.999

^*^Cancer recurrence within 24 months postoperatively. ^**^No recurrence after 24 months postoperatively. ^***^p-values could not be calculated because every patient had BRCA2 and TP53 mutations.

### Mutation allele frequency and phosphorylation analysis

**Figure 3:**
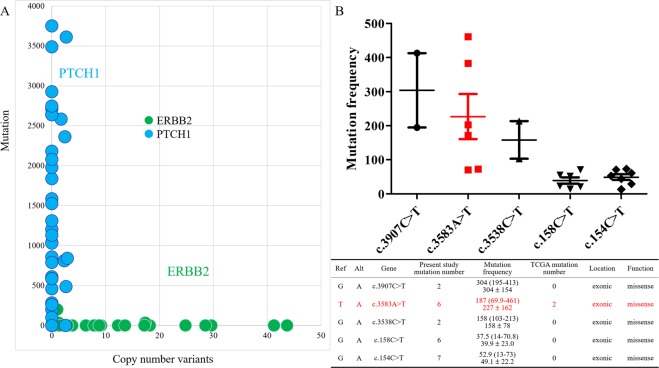
Mutation allele frequency and copy-number variation of *PTCH1* in breast cancer patients. (**A)** Comparison across 44 breast cancer samples by mutation allele frequency and copy-number variations (CNVs). *ERBB2* had a high CNV rate but low mutant rate, whereas *PTCH1* had a high mutation rate but an extremely low CNV rate in our study. (**B**) The upper graph shows the mutation frequencies of the top 5 mutation sites in *PTCH1*, including c.3907C > T, c.3583A > T, C.3538C > T, c.158C > T, and c.154C > T. Each dot represents an individual patient with a mutated gene. The mutation frequency was the product of allele frequency (AF) and read depth (DP). c.3583A > T had the highest mutation frequency and more patient number. The lower table shows detailed information. The mutation frequency is presented as median (range) and mean ± standard deviation. *PTCH1* mutation sites (Alt) were compared with the reference genome (Ref). The biological function and patient number of each mutated site in the present study and in the TCGA database are also listed.

The frequency of mutation alleles was compared with the copy number of each gene. Amplification of the *ERBB2* gene was detected in Her-2 (human epidermal growth factor receptor 2) enriched patients, and anti-Her-2 therapy was successfully applied to these patients. We used the *ERBB2* gene as a control. The *ERBB2* gene had a high rate of copy-number variations (CNV) and a particularly low mutation rate. However, *PTCH1* had particularly low CNVs but a surprisingly high mutation rate in these breast cancer patients (Fig. 3A). The details of *PTCH1* mutations are presented in Supplementary Fig. S2, and the most common 5 mutant sites on *PTCH1* were analyzed (Fig. 3B). The mutation frequency was the product of allele frequency (AF) and read depth (DP). *PTCH1* c.154C > T and c.158C > T (exon 1) were detected at a low frequency. We presumed that the number of cancer cells with *PTCH1* c.154C > T or c.158C > T was too small and the small number of mutated cells could not represent the whole heterogeneous cancer scenario. There were only two patients with *PTCH1* c.3907C > T (exon 23) and another two patients with c.3538C > T (exon 21) mutations. The patient number was insufficient to perform further analysis. We identified that c.3583A > T was the most frequent mutation in the present study and this mutation site was located in exon 22. Validation with Sanger sequencing was listed in Supplementary Table S3.

**Figure 4:**
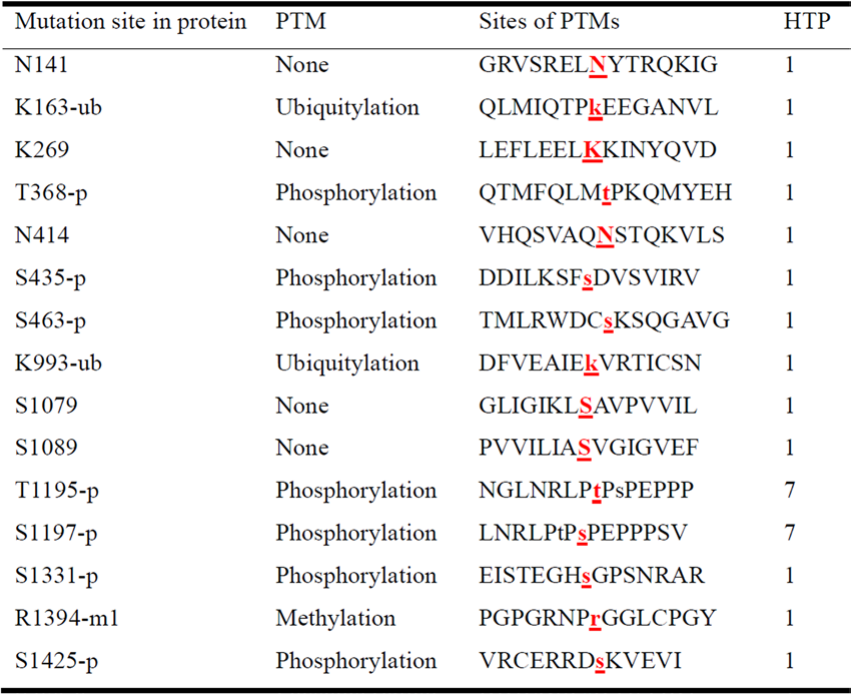
Posttranslational modification (PTM) sites of PTCH1 protein were identified by PhosphoSite. The PTMs in the proteins were located, and the below list focuses on the surrounding amino acid sequences. The targeted amino acid is marked with highlighted, red-colored, and bold. HTP, high-throughput publication.

**Figure 5:**
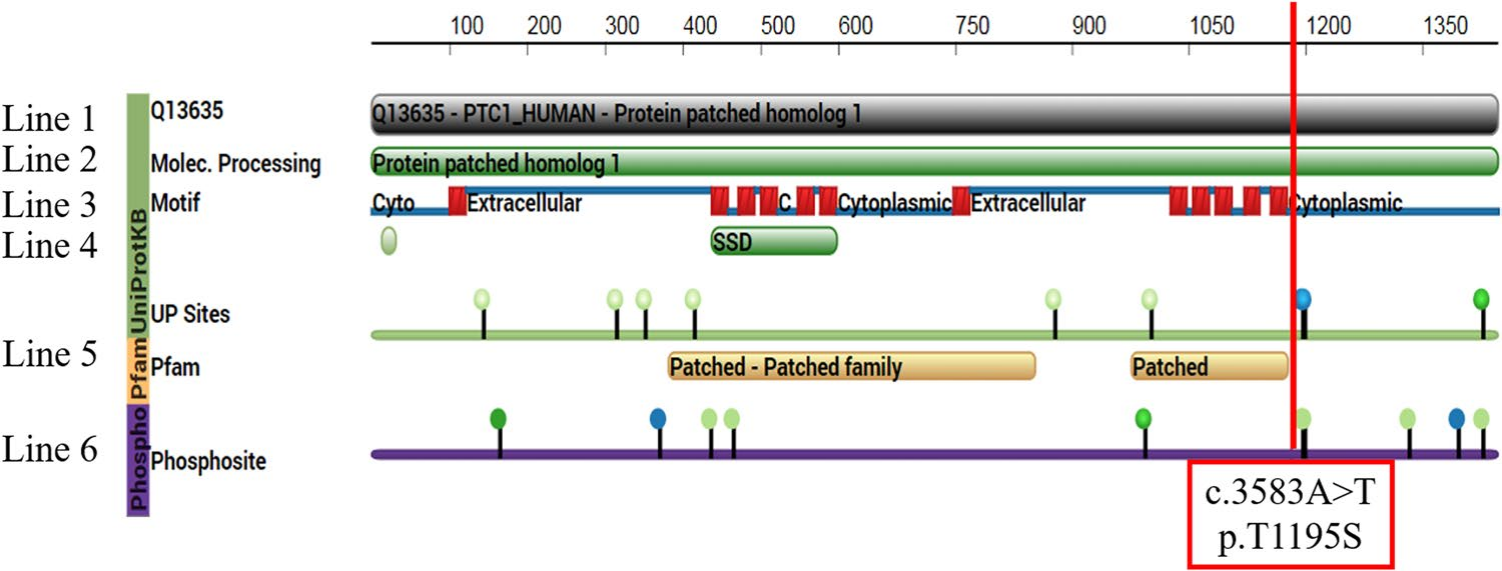
The alignment of the PTCH1 amino acid sequence of reference and the biology effect of the mutated sites for posttranslational modification. The 1^st^ line is the UniProt Knowledgebase (UniProtKB) sequence of human PTCH1 protein, entry number Q13635. The 2^nd^ line is the full sequence of PTCH1, patched homolog 1. The structural motif of PTCH1 protein is drawn in line 3. The transmembrane helices are shown as red boxes, and the extracellular and intracellular domains are shown as blue lines. The 4^th^ line is the sterol-sensing domain (SSD) for cholesterol signaling. The 5^th^ line is the binding site for ligand proteins (patched and patched family proteins). The 6^th^ line is the known sites of posttranslational modification. The green nodes are sites of phosphorylation, and the blue nodes are sites of methylation. c.3591C (phosphorylation of p.S1197 protein) is expressed as a green node. The mutation site c.3583A > T (p.T1195S in the protein) is marked with a red line and is located in the intracellular domain at the C-terminus of PTCH1 protein.

The highly mutated genes were correlated with protein phosphorylation through GO analysis (Fig. 1A). CLC Genomics Workbench was used to predict the impact of the *PTCH1* mutants on posttranslational modification (PTM). Our results were analyzed with a comprehensive resource of known phosphorylation sites, the PhosphoSite database^17^. More than half of the mutation sites showed novelty (Fig. 4). c.3583A > T occurred at the intracellular C-terminus and changed the amino acid from threonine to serine (mutation of amino acid: p.T1195S, Fig. 5). In the prediction model, c.3583A > T (p.T1195S) directly interrupted the protein kinase C phosphorylation (Fig. 4) and changed the protein structure (Supplementary Fig. S3). Multiple mutation sites located in exon 22 and exon 23 were predicted to affect phosphorylation, from c.3583A (p.T1195) to c.3992C (p.S1331) (Fig. 4). Based on the aforementioned results, we presumed that exons 22 and 23 were the potentially crucial segments of *PTCH1*.

### Mutations of the *PTCH1* gene in breast cancer are correlated with recurrence

**Figure 6:**
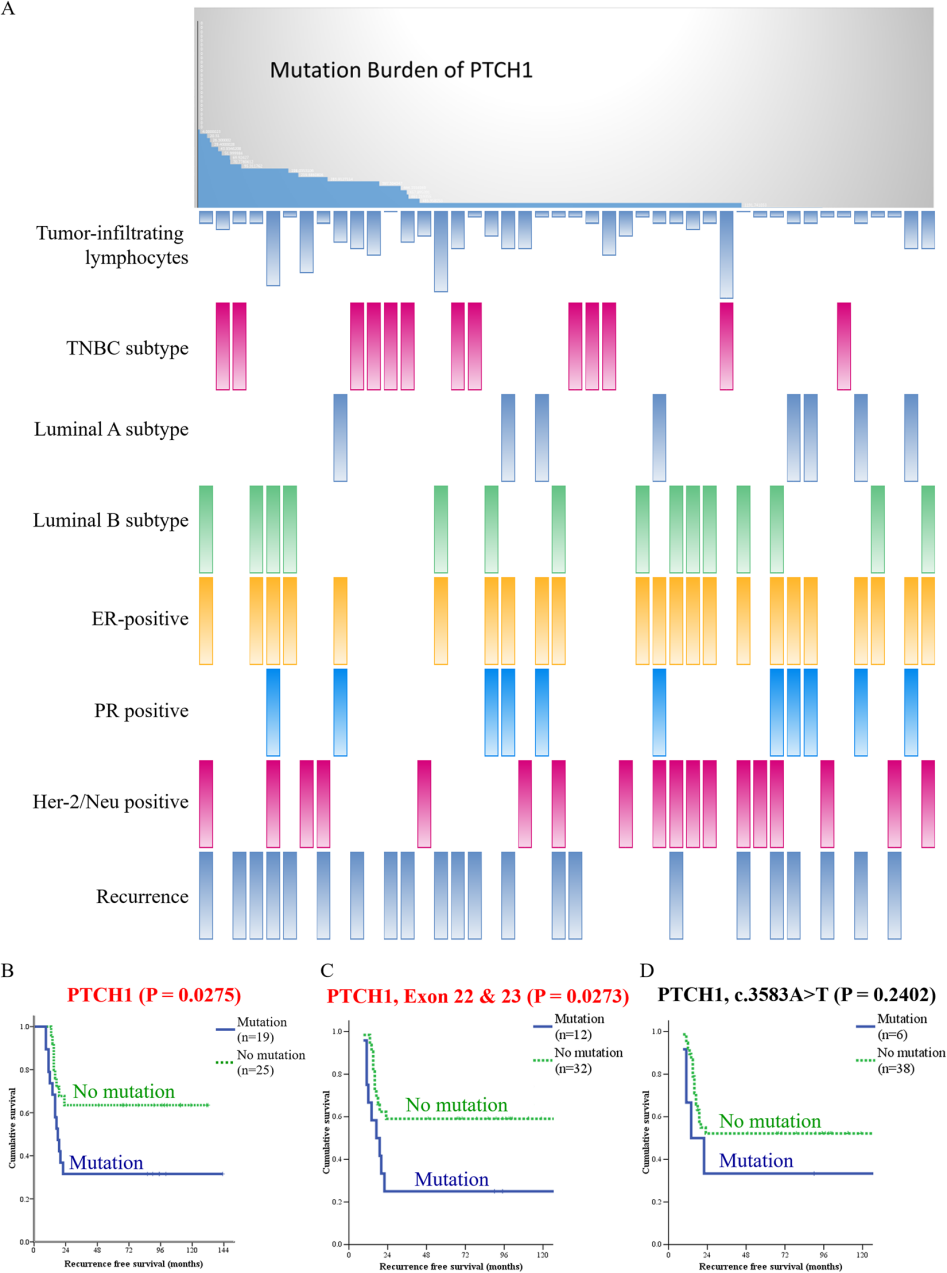
Mutations of *PTCH1* in breast cancer patients were correlated with poor survival and a high recurrence rate. (**A**) The upper graph shows the mutation burden of *PTCH1* from the current study; each bar represents an individual patient. The lower one represents the PheWAS analysis of the correlation between mutation of *PTCH1* and clinical outcomes. Each bar represents a single patient. A high mutation burden of *PTCH1* was correlated with a high rate of recurrence. (**B–D**) Kaplan-Meier analysis of recurrence-free survival according to the different mutation sites. (**B**) The patients were stratified into no mutation and all *PTCH1* mutations. (**C**) The patients were stratified into those without and with mutations in exons 22 & 23 of *PTCH1*. (**D**) The patients were stratified into without and with the *PTCH1* c.3583A > T mutation.

Mutations of *PTCH1* were correlated with demographics, histopathological findings and intrinsic subtypes (Table 4). Decreased amounts of tumor-infiltrating lymphocytes (TILs) were detected in breast cancer samples with mutated *PTCH1* (21% low TILs in mutated *PTCH1* vs. 79% low TILs in wild-type). A higher ratio of *PTCH1* mutations was found in luminal B breast cancer without Her-2/neu overexpression (67%) and triple-negative breast cancer (TNBC, 62%) compared with other subtypes (Table 4). The *PTCH1* mutation burden was correlated with TILs, intrinsic subtypes, prognostic predictors, and cancer recurrence by PheWAS analysis (Fig. 6A). The patients with mutated *PTCH1* had a higher ratio of metastasis in the lung, liver, and distant lymph nodes (Table 5). The patients with *PTCH1* mutations had poor recurrence-free survival (P = 0.0275, Figs 2 and 6B). Multivariate analysis of recurrence-free survival showed that the N3 nodal stage and *PTCH1* mutation strongly predicted recurrence (Table 6). *PTCH1* is a long gene with a total of 23 exons. To better understand the effect of *PTCH1* mutation on the clinical outcome of patients, we analyzed patients according to the exon harboring the mutation or to the specific mutation site (Supplementary Table S4 and Supplementary Fig. S4). Only one patient had a *PTCH1* mutation in exon 10 or 12, and no solid conclusion was obtained. *PTCH1* mutations in exons 3, 7, 15, and 21 were not correlated with survival. *PTCH1* mutations in exons 1, 6, 8, 14, 17, 19, 20, and 22 led to poor prognosis, though without statistical significance. The patients with mutated exon 23 of *PTCH1* had poor survival (P = 0.0179, Supplementary Fig. S4O). Mutation in the union of mutations in exons 22 and 23 predicted worse survival (P = 0.0273, Fig. 6C). *PTCH1* c.3583A > T (exon 22) mutations were correlated with poor patient survival, though the *p*-value was not statistically significant due to the small patient number (number of mutated patients = 6, Fig. 6D). Meanwhile, multiple mutation sites, from c.3583A (p.T1195) to c.3992C (p.S1331), located on exon 22 and exon 23, were predicted to affect phosphorylation (Fig. 4). All patients with lung or liver metastasis had mutations in exon 22, or 23 of *PTCH1*, which is in the C-terminus of the intracellular domain of PTCH1 protein (Supplementary Table S5). This result confirmed that exons 22 and 23 were potentially crucial segments of *PTCH1*.

**Table 4:** Correlation of wild-type or mutated *PTCH1* with demographics, histological findings, and intrinsic subtype in 44 patients with breast cancer who underwent radical resection and standard adjuvant therapy.

	Wild-type *PTCH1*	Mutated *PTCH1*	*p*-value
Patients, n (%)	25 (57%)	19 (43%)	
Age at surgery (years)*	47 (31–75)	50 (29–63)	0.454
Tumor size (cm)*	2.8 (1.5–6.0)	3.1 (1.4–8.0)	0.648
Nuclear grade			0.710
Grade II	6 (67%)	3 (33%)	
Grade III	19 (54%)	16 (46%)	
Extensive intraductal components	8 (53%)	7 (47%)	0.759
Lymphatic tumor emboli	13 (52%)	12 (48%)	0.547
Tumor-infiltrating lymphocytes			0.058
Low	11 (79%)	3 (21%)	
High	14 (47%)	16 (53%)	
Skin invasion	2 (67%)	1 (33%)	>0.999
Nipple invasion	3 (43%)	4 (57%)	0.432
Axillary lymph node metastasis			0.761
Negative	10 (53%)	9 (47%)	
Positive	15 (60%)	10 (40%)	
Positive lymph node numbers	1 (0–17)	1 (0–43)	0.853
Total resected lymph node numbers	19 (1–38)	21 (5–45)	0.297
Extranodal extension	3 (33%)	6 (67%)	0.257
Nodal staging			0.933
N0	10 (53%)	9 (47%)	
N1	8 (62%)	5 (38%)	
N2	5 (63%)	3 (37%)	
N3	2 (50%)	2 (50%)	
Tumor stage			0.906
T1	2 (67%)	1 (33%)	
T2	21 (57%)	16 (43%)	
T3	2 (50%)	2 (50%)	
AJCC TNM stage			0.921
Stage I	2 (67%)	1 (33%)	
Stage II	16 (55%)	13 (45%)	
Stage III	7 (58%)	5 (42%)	
Estrogen receptor			0.361
Negative (<10%)	10 (48%)	11 (52%)	
Positive (≥ 10%)	15 (65%)	8 (35%)	
Progesterone receptor			0.474
Negative (<10%)	18 (53%)	16 (47%)	
Positive (≥ 10%)	7 (70%)	3 (30%)	
Her-2/Neu			0.124
Negative	12 (46%)	14 (54%)	
Positive	13 (72%)	5 (28%)	
Intrinsic subtype			0.192
Luminal A	6 (75%)	2 (25%)	
Luminal B without Her-2/Neu overexpression	2 (33%)	4 (67%)	
Luminal B with Her-2/Neu overexpression	7 (78%)	2 (22%)	
Her-2/Neu overexpression	5 (63%)	3 (37%)	
Triple-negative breast cancer	5 (38%)	8 (62%)	

AJCC TNM stage, American Joint Committee on Cancer tumor-node-metastases (TNM) staging system, 7^th^ ed.

*Values are expressed as median (range).

**Table 5:** Correlation between cancer recurrence and wild-type or mutated PTCH1 in patients with breast cancer who underwent radical resection and standard adjuvant therapy.

	Wild-type PTCH1	Mutated PTCH1	*p*-value
Patients, n (%)	25 (57%)	19 (43%)	
Recurrence	9 (41%)	13 (59%)	0.067
Lung metastasis	5 (31%)	11 (69%)	0.013^*^
Liver metastasis	2 (20%)	8 (80%)	0.011^*^
Bone metastasis	6 (40%)	9 (60%)	0.123
Brain metastasis	3 (50%)	3 (50%)	>0.999
Regional lymph node recurrence	2 (40%)	3 (60%)	0.638
Distant lymph node metastasis	4 (31%)	9 (69%)	0.044^*^
Local recurrence	0	2 (100%)	0.181
Skin metastasis	1 (50%)	1 (50%)	>0.999
Adrenal gland metastasis	1 (50%)	1 (50%)	>0.999
Splenic metastasis	1 (33%)	2 (67%)	0.570

^*^p-value less than 0.05.

**Table 6:** Multivariate analysis of hazard ratio according to clinicopathological factors and *PTCH1* mutation in patients with breast cancer (n = 44).

	Hazard radio	95% Confidence interval	*p*-value
Age at surgery (years)	0.973	0.925–1.023	0.280
Nodal stage^*^			0.007^**^
N0	1		
N1	0.793	0.274–2.295	0.669
N2	0.589	0.147–2.360	0.455
N3	7.799	1.940–31.353	0.004^**^
*PTCH1* gene			0.010^**^
Wild-type *PTCH1*	1		
Mutated *PTCH1*	3.453	1.344–8.870	

^*^According to American Joint Committee on Cancer tumor-node-metastasis (TNM) staging system, 7^th^ ed. ^**^p-value less than 0.05.

## Discussion

The breakthroughs of the Human Genomic Project have accelerated the search for somatic mutations that drive the initiation and progression of human cancers. A combination of public databases of online information and targeted deep sequencing of 44 breast cancer patients in National Cheng Kung University Hospital identified *PTCH1* mutations in the present study. Mutation of *PTCH1* was predicted to correlate with protein phosphorylation. The cancer samples with mutated *PTCH1* had a lower number of tumor-infiltrating lymphocytes. The patients with mutated *PTCH1* had more metastasis in the lung, liver or distant lymph nodes. The patients with mutated *PTCH1*, especially exons 22 and 23, had a worse recurrence-free survival. These findings suggest that mutation of *PTCH1* is a powerful predictor for breast cancer patients and the exons 22 and 23 are potentially vital regions.

Previous studies emphasize that the few mutations occur at a high frequency within or across cancer types; however, most mutations in cancer genomes occur infrequently. These rare mutations collectively play a defining role in one-fourth of all human cancers. High-frequency mutations drive critical molecular, biological and clinical phenotypes, while rare mutations promote the development of precision medicine^10^. Somatic mutations spontaneously occur in the body’s cells and accumulate throughout a lifetime, which may contribute to cancer and other diseases^18,19^. In contrast, germline mutations are present in gametes and passed to the next generation by inheritance^20^. Advancements in genome sequencing techniques and large-scale databases have revolutionized our knowledge of the mutations in cancer. When genetic mutation yields a clone of cells that circumvent the restrictions of normal cells, it will further promote cancer development and progression^21,22^.

The present study identified mutations in 44 breast cancer patients in Taiwan. A total of 143 cancer-related genes were sequenced. We also used bioinformatics approaches to identify potential mutations in breast cancer and predict their effects. Identifying genomic signatures in breast cancer patients will provide a better understanding of further clinical applications. Based on our results, the most frequent mutations were identified in *BRCA2*, *APC*, *ATM*, *BRCA1*, *NF1*, *TET2*, *TSC1*, *TSC2*, *NOTCH1*, *MSH2*, *PTCH1*, *TP53*, *PIK3CA*, *FBXW7*, and *RB1* (Fig. 1B and Supplementary Fig. S1). Our data identified different incidence of mutation in these genes. All patients had mutations in the *BRCA2* and *TP53* genes. The clinical significance of these mutations requires further study. Other patients with gene mutations had similar recurrence-free survival to those without mutations. The patients with the *PTCH1* mutation had a worse recurrence-free survival than those with wild-type *PTCH1* (Fig. 2). High mutated allele frequency and low copy-number variation of *PTCH1* was a unique presentation (Fig. 3A). According to our data, the function of *PTCH1* in breast cancer was presumed to depend on mutation frequency, not the expression level. Based on the aforementioned results, *PTCH1* was chosen for further study.

*PTCH1* gene encodes the patched homolog 1 (PTCH1) protein. PTCH1, a 12-pass transmembrane protein, contains two large extracellular loops and two large intracellular loops. PTCH1 protein is one of the membranous receptors in Hedgehog signaling^23,24^. The sterol-sensing domain (SSD) is the segment involved in cholesterol synthesis and transport. Hedgehog signaling is central to the embryonic development of various tissues and organs. An increasing number of tumors arise due to mutations in this pathway, such as basal cell carcinoma (BCC) of the skin^25^. Many studies have focused on the function of PTCH1 protein in human disorders and tumorigenesis^25–27^. The extracellular loop of PTCH1 protein interacts with sonic hedgehog (SHH) or desert hedgehog (DHH) protein. Without ligand binding, PTCH1 protein inhibits Smoothened (SMO) receptors and inactivates downstream pathways. Binding of SHH/DHH protein with PTCH1 receptor activates the SMO coreceptor and downstream GLI1 signaling. Without the presence of hedgehog ligand, PTCH1 receptor has other roles in the cell, such as restricting the range of signaling, inhibiting cell cycle progression by sequestering cyclin B1, or mediating cell apoptosis via caspase 3- and caspase 9-related signaling pathways^28,29^. The PTCH1 receptor is supposed to be a tumor suppressor. The Hedgehog signaling pathway potentially activates breast carcinogenesis by increasing the expression of SHH, SMO and GLI1 proteins and reducing the expression of PTCH1^30^. However, overexpression of PTCH1 protein is detected in breast cancer, especially in luminal B and TNBC subtypes^31^. While overexpression of PTCH1 protein is detected in many kinds of cancer, the function of PTCH1 changes from tumor suppressor to drug transporter for chemotherapeutic agents. PTCH1 protein uses the proton-motive force and establishes a reversed pH gradient to efflux drugs, such as doxorubicin. The GXXXD motif of putative transmembrane segment 4 is highly conserved in humans and drosophila. The glycine residue at position 509 and the aspartic acid residue at position 513 are important in drug efflux^32^. In cancer cell lines from adrenocortical carcinoma or melanoma, doxorubicin efflux is handled by overexpressing the PTCH1 protein^33,34^. PTCH1 is a potential therapeutic target for PTCH1-overexpressing lung, breast, prostate, ovary, colon, brain, adrenocortical carcinoma and melanoma^35^. However, these studies focus on overexpression of PTCH1 protein, not sequencing of the *PTCH1* gene. Overexpression of a past-identified tumor suppressor in cancer tissue potentially implicates an alteration of protein structure and function. Mutation of the *PTCH1* gene may function as the turning point from tumor suppressor to drug transporter.

Mutations of the *PTCH1* gene are believed to be responsible for the development of both sporadic and familial BCCs^24,26^. However, there is a lack of research on *PTCH1* mutations in breast cancer. Based on the results of next-generation sequencing, the polymorphism of *PTCH1* c. 3944C > T (p.P1315L) is associated with the incidence of breast cancer after the use of oral contraceptives^36^. We excluded *PTCH1* c. 3944C > T because of the high prevalence of this polymorphism and the low usage of oral contraceptives in Taiwan. We conducted deep targeted sequencing of the *PTCH1* gene from 44 breast cancer patient samples. The samples with mutated *PTCH1* contained a lower number of TILs (Table 4). The patients with *PTCH1* mutations had a higher ratio of lung, liver, or distant lymph node metastasis (Table 5) and worse recurrence-free survival (Figs 2 and 6B). After analysis of all mutation sites on *PTCH1*, c.3583 A > T was the highest mutation point (Fig. 3B). The patients with mutated exon 22 and exon 23 of *PTCH1* or mutated exon 23 had poor prognosis (Fig. 6C and Supplementary Fig. S4O). The clinical importance of *PTCH1* mutations in breast cancer is implicated.

We found that all highly mutated genes were correlated with protein phosphorylation, such as peptidyl-tyrosine phosphorylation, protein phosphorylation, and protein autophosphorylation (Fig. 1A). We tried to analyze *PTCH1* mutations in protein structure and function. The PhosphoSite database showed that half of the mutation sites in *PTCH1* genes can alter posttranslational modifications of the protein (Fig. 4). CLC Genomics Workbench was used to predict the protein structure of the *PTCH1* c.3583A > T mutant (Supplementary Fig. S3). *PTCH1* c.3583A (p.T1195) mutation interrupted protein phosphorylation (Fig. 5). Multiple mutation sites, from c.3583A to c.3992C (p.S1331), located in exon 22 and exon 23, were predicted to affect phosphorylation (Fig. 4). Exons 22 and 23 encode parts of the intracellular C-terminus of the PTCH1 protein and interact with the SMO receptor. Blockage of the interaction between PTCH1 and SMO will retrain signal transduction and suppress GLI1 protein expression. Exons 22 and 23 encode potentially crucial segments for PTCH1 protein in breast cancer. Although further efforts should be applied to understand the role of PTCH1 in breast cancer, we believe that the present study suggests unprecedented value in assessing PTCH1 in breast cancer patients. Future research can be based on these cancer susceptibility genes in this study and tease out their roles in breast cancer.

Our study also had some limitations because of the low sample numbers, with only 44 patients, and the risk of DNA damage in formalin-fixed paraffin-embedded (FFPE) samples. Most breast cancer patients have a better survival after standard treatment and seldom develop recurrence within 24 months postoperatively. The patients with the worst outcome were rare, and this was the reason that the present study had to run from 2006 to 2017 to let us collect 22 recurrent patients. A previous study indicated that the sensitivity and specificity to detect mutations in FFPE samples were reliable when comparing next-generation sequencing assays with Oncomine Cancer Panel assays^37^. The damage of the DNA template in FFPE samples was a possible source of sequencing artifacts^38^. We tried to eliminate artifacts with improved DNA extraction and PCR amplification techniques. Sanger sequencing was also used to confirm the mutation sites. However, the possibility of artifacts could not be completely excluded, and further study of *PTCH1* mutations in breast cancer patients is necessary.

## Conclusions

We performed targeted deep sequencing of 143 cancer-related genes for 44 breast cancer samples. Genomic mutation and copy-number variations were analyzed and compared with the data in public databases. The functions of mutated genes were predicted by GO analysis, the PhosphoSite database, and CLC Genomics Workbench. We detected the *PTCH1* mutation in 18 of 44 patients, and c.3583A > T had the highest mutation frequency. Multiple mutation sites from c.3583A to c.3992C located in exon 22 and exon 23 of *PTCH1* were predicted to affect phosphorylation. The patients with mutated *PTCH1* had a high risk of metastasis in the lung, liver, or distant lymph nodes and a decreased number of TILs in breast cancer samples. The patients with mutated exon 23 or mutated exons 22 and 23 of *PTCH1* had poor survival. These data suggest that exons 22 and 23 are crucial for *PTCH1* and that *PTCH1* can be a powerful predictor for the recurrence of breast cancer patients.

## Materials and Methods

### Patients and targeted deep sequencing for mutation detection

Formalin-fixed, paraffin-embedded (FFPE) tissue specimens from breast cancer patients were collected from the Department of Pathology in the National Cheng Kung University Hospital (NCKUH). Written informed consent was collected for all patients, and the Institutional Review Board of NCKUH approved this study under NCKUH IRB number A-ER-105-233. The study also complied with the formal guidelines of the Institutional Review Board. The collection period was from April 2006 to January 2017. A total of 22 patients were defined as the recurrence group, having cancer recurrence within 24 months postoperatively. Another 22 matched patients without cancer recurrence after surgery and standard treatment were selected as the control group. All these patients received radical operation and standard adjuvant therapy. Patient information was collected from a retrospective chart reviewing demographics, histopathologic findings, and clinical results as we previously described^39,40^. The TNM (tumor, node, metastasis) stage was defined according to the American Joint Committee on Cancer (AJCC) classification of 2010, 7^th^ edition (corrected printing 2015)^41^.

DNA and RNA were extracted from these cancer tissues. Sufficient amount of tissue was used to extract DNA/RNA after removal of formaldehyde-induced crosslinks by heat and removal of protein-DNA crosslinks by proteinase K. Fluorometry was used to assess quality of double-stranded DNA. Short amplicons of DNA was generated to increase number of templates for PCR and specific primers for each strand was applied in amplicon-based target enrichment. RNA was reverse-transcribed to make cDNA, and target regions from DNA or cDNA were amplified by the DNA/RNA Oncomine Cancer Research Panel version 1 (Thermo Fisher Scientific, Waltham, USA). High-fidelity DNA polymerase was used to reduce polymerase errors and bypass uracil/abasic sites. An Ion 318 chip (Thermo Fisher Scientific) was prepared and loaded according to the manufacturer’s recommendation. The Ion PGM Sequencing 200 Kit v.2 (Thermo Fisher Scientific) was used with the Ion PGM sequencer (Thermo Fisher Scientific) as described in the Ion PGM Sequencing Kit User Guide. The sequencing reads were aligned to the reference genome (hg19) and variants called by Torrent Suite 5.0.4^42,43^. The sequencing data of 44 breast cancer samples were available for analysis of 143 genes (Supplementary Table S1). Variant calling was conducted using a locked data analysis pipeline, Torrent Suite version 5.2.2 (Thermo Fisher Scientific) and Oncomine Comprehensive w2.3 - DNA and Fusions - Single Sample v5.6 in Ion Reporter version 5.6 (Thermo Fisher Scientific). The minimal depth of single-nucleotide variants (SNVs) and small insertions/deletions (indels) was 15. The minimal allele frequency (AF) was 7% for indels. Some SNV hotspots were designed in the Oncomine system, and the minimal AF was 3% for SNVs in hotspots. For SNVs not in hotspots, the minimal AF was 4%.

Whole-genome sequencing data of 499 normal Taiwanese were provided by Taiwan Biobank to compare the distribution and frequency of different genes between cancer patients and the normal population as previously described^44^. Publicly available data were used to compare and validate the distribution of the *PTCH1* genetic variant worldwide. Phenome-wide association scans (PheWAS) were used to link the characteristics of cancer and mutations of PTCH1.

### Sanger sequencing for validation of mutation

To confirm the variant detection analysis, we used Sanger sequencing for the high-frequency variants. PCR amplification and DNA sequencing primers were designed and synthesized by Mission Biotech, Taipei, Taiwan. The primer sequences for the *PTCH1* gene were as follows: *PTCH1*-F: TGA CAC TGT CGT CTG GGA AC; *PTCH1*-R: AAC AGA GGC CCC TGA AAA AT. For the PCR amplification, the target regions were amplified using the KAPA HiFi HotStart PCR kit (KAPA Biosystems, Wilmington, USA) in a total reaction volume of 50 μL. Reactions were run in a 9700-thermal cycler (Applied Biosystems, Waltham, USA) using the following cycling parameters: 3 min holding at 95 °C, followed by 25 cycles of 20 sec at 95 °C, 20 sec at 66 °C, and 30 sec at 72 °C. The presence of amplicons was confirmed by gel electrophoresis on a 1.5% agarose gel. The PCR Fragment Extraction Kit (Geneaid, New Taipei, Taiwan) was used for the PCR amplicon purification process. DNA sequencing was done by using the ABI PRISM BigDye Terminator Cycle Sequencing Ready Reaction Kit v3.1 (Applied Biosystems) on the ABI Prism 3730XL DNA Analyzer. Sanger chromatograms were analyzed using CLC Genomics Workbench, Aarhus, Denmark.

### Gene ontology cluster and analysis

Clustering of Gene Ontology (GO) terms furnishes groups of genes sharing similar functions or characteristics. Based on standard GO terms, comparative genomic studies in different diseases are made possible^45,46^. GO term analysis begins with three main features: biological process, molecular function, and cellular component^47^. The GO list was generated from the CLC Genomics Workbench by analyzing mutation patterns in 44 breast cancer patients. Afterward, sequences from these patients were grouped with GO terms using BLAST2GO PRO plugin 1.1.0 within CLC Genomics Workbench. A hypergeometric distribution test was used to link the association between 143 genes and GO class. The Benjamini-Hochberg procedure was used to avoid type I errors and decrease the false discovery rate. A corrected *p*-value cutoff <0.05 was set. Finally, the output results were processed on the REViGO web-server tool to remove redundant GO terms (http://revigo.irb.hr/). REViGO has many features for postanalysis of GO terms that connect to third-party plug-ins, such as Cytoscape Scatterplots and Tree Mapping^48^.

### Statistical analysis

All statistical results were conducted using SPSS version 17.0 (SPSS, New York, USA). The continuous variables are reported as median (range) and were compared using the nonparametric Kruskal-Wallis H test. Fisher’s exact test and the chi-square test were used to compare the differences in categorical variables. Kaplan-Meier survival analysis and the log-rank test were used to estimate patient survival and quantify the differences between groups. Those factors with P < 0.2 in the univariate analysis were included in the multivariate analysis. A Cox proportional hazards regression model was used to analyze significant factors in recurrence-free survival in multivariate analysis. The results are expressed as the hazard ratio (HR) with 95% confidence interval (95% C.I.). The *p*-value cut-off was set at 0.05 for statistically significant differences.

## Supplementary information

Supplementary Dataset 1
