# Supplementary material for: Mutation of the PTCH1 gene predicts recurrence of breast cancer
Source: Sci Rep. 2019 Nov 8;9:16359. doi: 10.1038/s41598-019-52617-4 (PMC6841698; doi:10.1038/s41598-019-52617-4)

Research Article:

Mutation of the *PTCH1* gene predicts recurrence of breast cancer

Chih-Yang Wang, PhD[1,2], Yung-Chieh Chang, MS[1], Yao-Lung Kuo, MD PhD[3], Kuo-Ting Lee, MD[3], Pai-Sheng Chen, PhD[1,4], Chun Hei Antonio Cheung, PhD[1,2], Chih-Peng Chang, PhD[1,5], Nam Nhut Phan, MS[6], Meng-Ru Shen, MD PhD[2,7], Hui-Ping Hsu, MD PhD[3]*

[1]Institute of Basic Medical Sciences, College of Medicine, National Cheng Kung University, Tainan, Taiwan

[2]Department of Pharmacology, College of Medicine, National Cheng Kung University, Tainan, Taiwan

[3]Department of Surgery, National Cheng Kung University Hospital, College of Medicine, National Cheng Kung University, Tainan, Taiwan

[4]Department of Medical Laboratory Science and Biotechnology, College of Medicine, National Cheng Kung University, Tainan, Taiwan

[5]Department of Microbiology and Immunology, College of Medicine, National Cheng Kung University, Tainan, Taiwan

[6]NTT Institute of Hi-Technology, Nguyen Tat Thanh University, Ho Chi Minh City, Vietnam

[7]Department of Obstetrics and Gynecology, National Cheng Kung University Hospital, College of Medicine, National Cheng Kung University, Tainan, Taiwan

*Corresponding author:

Hui-Ping Hsu, Department of Surgery, National Cheng Kung University Hospital, College of Medicine, National Cheng Kung University, Tainan, Taiwan

Address: No. 138, Sheng-Li Rd. Tainan City, 704, Taiwan

E-mail: hphsu@mail.ncku.edu.tw

Tel.: +886-6-2353535#5272

Fax: +886-6-2766676

Supplementary Table S1. Gene lists in Oncomine Comprehensive Assays version 1 (ThermoFisher®). Total 143 genes were targeted, including 73 genes with hotspot mutations, 49 genes with focal CNV (copy number variants) gains, 26 genes with full CDS (coding DNA sequences) for DEL mutations and CNV loss, and 22 genes with fusion drivers. All these target genes were used in different applications, including 9 genes on 31 Labels, 12 genes in 17 guidelines, 64 genes for drug targets and 64 genes in >800 global clinical trials.

Hotspot genes, n=73 (hotspot coverage)			Copy Number Variants			Fusion drives, n=22 (183 assays)
			CDS, n=26 (full gene)	Copy gain, n=49		
ABL1	GATA2	MYD88	APC	ACVRL1	IGF1R	ALK
AKT1	GNA11	NFE2L2	ATM	AKT1	IL6	RET
ALK	GNAQ	NPM1	BAP1	APEX1	KIT	ROS1
AR	GNAS	NRAS	BRCA1	AR	KRAS	NTRK1
ARAF	HNF1A	PAX5	BRCA2	ATP11B	MCL1	ABL1
BRAF	HRAS	PDGFRA	CDH1	BCL2L1	MDM2	AKT3
BTK	IDH1	PIK3CA	CDKN2A	BCL9	MDM4	AXL
C15orf23	IDH2	PPP2R1A	FBXW7	BIRC2	MET	BRAF
CBL	IFITM1	PTPN11	GATA3	BIRC3	MYC	CDK4
CDK4	IFITM3	RAC1	MSH2	CCND1	MYCL	ERBB2
CHEK2	JAK1	RAF1	NF1	CCNE1	MYCN	ERG
CSF1R	JAK2	RET	NF2	CD274	MYO18A	ETV1
CTNNB1	JAK3	RHEB	NOTCH1	CD44	NKX2-1	ETV4
DDR2	KDR	RHOA	PIK3R1	CDK4	NKX2-8	ETV5
DNMT3A	KIT	SF3B1	PTCH1	CDK6	PCD1LG2	FGFR1
EGFR	KRAS	SMO	PTEN	CSNK2A1	PDGFRA	FGFR2
ERBB2	MAGOH	SPOP	RB1	DCUN1D1	PIK3CA	FGFR3
ERBB3	MAP2K1	SRC	SMAD4	EGFR	PNP	NTRK3
ERBB4	MAP2K2	STAT3	SMARCB1	ERBB2	PPARG	PDGFRA
ESR1	MAPK1	U2AF1	STK11	FGFR1	RPS6KB1	PPARG
EZH2	MAX	XPO1	TET2	FGFR2	SOX2	RAF1
FGFR1	MED12		TP53	FGFR3	TERT	
FGFR2	MET		TSC1	FGFR4	TIAF1	
FGFR3	MLH1		TSC2	FLT3	ZNF217	
FLT3	MPL		VHL	GAS6		
FOXL2	MTOR		WT1			

Supplementary Table S2. Predicting gene ontology biological process from mutation data. The mutation statues carried out based on GO annotation and analysis. Enrichment of functionally related GO terms among the mutation gene and clustering of significant GO term was analyzed by CLC genomic workbench (full list).

GO	term	Description	*p*-values
GO:	0007155	cell adhesion	1.53E-14
GO:	0016477	cell migration	8.63E-11
GO:	0030198	extracellular matrix organization	1.01E-10
GO:	0018108	peptidyl-tyrosine phosphorylation	1.21E-08
GO:	0006468	protein phosphorylation	2.38E-08
GO:	0007411	axon guidance	3.83E-08
GO:	0030036	actin cytoskeleton organization	3.99E-08
GO:	0046777	protein autophosphorylation	5.64E-08
GO:	0007507	heart development	6.76E-07
GO:	0051056	regulation of small GTPase mediated signal transduction	6.99E-07
GO:	0048010	vascular endothelial growth factor receptor signaling pathway	7.79E-07
GO:	2000463	positive regulation of excitatory postsynaptic potential	2.34E-06
GO:	0009968	negative regulation of signal transduction	2.69E-06
GO:	0071300	cellular response to retinoic acid	4.30E-06
GO:	0007165	signal transduction	8.22E-06
GO:	0043410	positive regulation of MAPK cascade	1.21E-05
GO:	0007399	nervous system development	1.34E-05
GO:	0010595	positive regulation of endothelial cell migration	1.76E-05
GO:	0006024	glycosaminoglycan biosynthetic process	1.98E-05
GO:	0060997	dendritic spine morphogenesis	2.64E-05
GO:	0048013	ephrin receptor signaling pathway	3.01E-05
GO:	0018105	peptidyl-serine phosphorylation	4.74E-05
GO:	0035556	intracellular signal transduction	5.43E-05
GO:	0001954	positive regulation of cell-matrix adhesion	6.52E-05
GO:	0048565	digestive tract development	6.52E-05
GO:	0043542	endothelial cell migration	6.76E-05
GO:	0033674	positive regulation of kinase activity	7.83E-05

GO:	0052696	flavonoid glucuronidation	7.83E-05
GO:	0090129	positive regulation of synapse maturation	7.83E-05
GO:	0045165	cell fate commitment	8.00E-05
GO:	0001843	neural tube closure	9.64E-05
GO:	0042059	negative regulation of epidermal growth factor receptor signaling pathway	9.64E-05
GO:	2000145	regulation of cell motility	9.64E-05
GO:	0038083	peptidyl-tyrosine autophosphorylation	1.12E-04
GO:	0008285	negative regulation of cell proliferation	1.13E-04
GO:	0007416	synapse assembly	1.17E-04
GO:	0051968	positive regulation of synaptic transmission, glutamatergic	1.18E-04
GO:	0000165	MAPK cascade	1.20E-04
GO:	0045893	positive regulation of transcription, DNA-templated	1.30E-04
GO:	0010976	positive regulation of neuron projection development	1.51E-04
GO:	0071526	semaphorin-plexin signaling pathway	1.56E-04
GO:	0070374	positive regulation of ERK1 and ERK2 cascade	1.64E-04
GO:	0070848	response to growth factor	1.92E-04
GO:	0007420	brain development	2.19E-04
GO:	0007158	neuron cell-cell adhesion	2.39E-04
GO:	0070102	interleukin-6-mediated signaling pathway	2.39E-04
GO:	0007275	multicellular organism development	2.52E-04
GO:	0043124	negative regulation of I-kappaB kinase/NF-kappaB signaling	2.57E-04
GO:	0007417	central nervous system development	2.67E-04
GO:	0030534	adult behavior	2.82E-04
GO:	0009887	animal organ morphogenesis	2.88E-04
GO:	0052697	xenobiotic glucuronidation	3.08E-04
GO:	0030335	positive regulation of cell migration	3.54E-04
GO:	0006897	endocytosis	3.67E-04
GO:	0030154	cell differentiation	3.88E-04
GO:	0072659	protein localization to plasma membrane	4.30E-04
GO:	2000311	regulation of alpha-amino-3-hydroxy-5-methyl-4-isoxazole propionate selective glutamate receptor	4.60E-04

		activity	
GO:	0018107	peptidyl-threonine phosphorylation	5.61E-04
GO:	0003148	outflow tract septum morphogenesis	5.92E-04
GO:	0019933	cAMP-mediated signaling	5.92E-04
GO:	0050808	synapse organization	5.92E-04
GO:	0045669	positive regulation of osteoblast differentiation	6.38E-04
GO:	0046328	regulation of JNK cascade	7.21E-04
GO:	0032147	activation of protein kinase activity	7.22E-04
GO:	0061024	membrane organization	7.53E-04
GO:	0042297	vocal learning	8.00E-04
GO:	0043535	regulation of blood vessel endothelial cell migration	8.00E-04
GO:	0051271	negative regulation of cellular component movement	8.00E-04
GO:	0061298	retina vasculature development in camera-type eye	8.00E-04
GO:	0007612	learning	8.13E-04
GO:	0007169	transmembrane receptor protein tyrosine kinase signaling pathway	8.48E-04
GO:	0003180	aortic valve morphogenesis	8.66E-04
GO:	0071625	vocalization behavior	8.91E-04
GO:	2000310	regulation of N-methyl-D-aspartate selective glutamate receptor activity	8.91E-04
GO:	0001934	positive regulation of protein phosphorylation	9.08E-04
GO:	0007010	cytoskeleton organization	9.46E-04
GO:	0045765	regulation of angiogenesis	1.00E-03
GO:	0007229	integrin-mediated signaling pathway	1.01E-03
GO:	0010863	positive regulation of phospholipase C activity	1.04E-03
GO:	0001764	neuron migration	1.05E-03
GO:	0007157	heterophilic cell-cell adhesion via plasma membrane cell adhesion molecules	1.05E-03
GO:	0045732	positive regulation of protein catabolic process	1.06E-03
GO:	2000641	regulation of early endosome to late endosome transport	1.07E-03

GO:	0007173	epidermal growth factor receptor signaling pathway	1.08E-03
GO:	0007179	transforming growth factor beta receptor signaling pathway	1.09E-03
GO:	0030032	lamellipodium assembly	1.15E-03
GO:	0030041	actin filament polymerization	1.15E-03
GO:	0045332	phospholipid translocation	1.15E-03
GO:	0006895	Golgi to endosome transport	1.18E-03
GO:	0045599	negative regulation of fat cell differentiation	1.27E-03
GO:	0060070	canonical Wnt signaling pathway	1.29E-03
GO:	0000122	negative regulation of transcription from RNA polymerase II promoter	1.43E-03
GO:	0006811	ion transport	1.47E-03
GO:	0090630	activation of GTPase activity	1.47E-03
GO:	0030168	platelet activation	1.49E-03
GO:	0030336	negative regulation of cell migration	1.58E-03
GO:	0001568	blood vessel development	1.62E-03
GO:	1904646	cellular response to beta-amyloid	1.62E-03
GO:	0035176	social behavior	1.91E-03
GO:	0045747	positive regulation of Notch signaling pathway	1.91E-03
GO:	0045944	positive regulation of transcription from RNA polymerase II promoter	1.93E-03
GO:	0031623	receptor internalization	2.00E-03
GO:	0008284	positive regulation of cell proliferation	2.04E-03
GO:	0050770	regulation of axonogenesis	2.25E-03
GO:	0006027	glycosaminoglycan catabolic process	2.29E-03
GO:	0007517	muscle organ development	2.67E-03
GO:	0014065	phosphatidylinositol 3-kinase signaling	2.70E-03
GO:	0002053	positive regulation of mesenchymal cell proliferation	2.74E-03
GO:	0045945	positive regulation of transcription from RNA polymerase III promoter	2.74E-03
GO:	0045956	positive regulation of calcium ion-dependent exocytosis	2.74E-03
GO:	0048009	insulin-like growth factor receptor signaling pathway	2.74E-03
GO:	0048705	skeletal system morphogenesis	2.74E-03

GO:	1900273	positive regulation of long-term synaptic potentiation	2.74E-03
GO:	0098609	cell-cell adhesion	3.08E-03
GO:	0001736	establishment of planar polarity	3.33E-03
GO:	0001945	lymph vessel development	3.33E-03
GO:	0009649	entrainment of circadian clock	3.33E-03
GO:	0043583	ear development	3.33E-03
GO:	0045176	apical protein localization	3.33E-03
GO:	0046903	secretion	3.33E-03
GO:	0051014	actin filament severing	3.33E-03
GO:	0060841	venous blood vessel development	3.33E-03
GO:	0071681	cellular response to indole-3-methanol	3.33E-03
GO:	0072207	metanephric epithelium development	3.33E-03
GO:	0097107	postsynaptic density assembly	3.33E-03
GO:	0097119	postsynaptic density protein 95 clustering	3.33E-03
GO:	0097151	positive regulation of inhibitory postsynaptic potential	3.33E-03
GO:	0007156	homophilic cell adhesion via plasma membrane adhesion molecules	3.37E-03
GO:	0030900	forebrain development	3.40E-03
GO:	0042058	regulation of epidermal growth factor receptor signaling pathway	3.40E-03
GO:	0060261	positive regulation of transcription initiation from RNA polymerase II promoter	3.40E-03
GO:	1990000	amyloid fibril formation	3.40E-03
GO:	2000650	negative regulation of sodium ion transmembrane transporter activity	3.40E-03
GO:	0007269	neurotransmitter secretion	3.59E-03
GO:	0051491	positive regulation of filopodium assembly	3.59E-03
GO:	0001558	regulation of cell growth	3.78E-03
GO:	0007492	endoderm development	3.84E-03
GO:	0010763	positive regulation of fibroblast migration	3.84E-03
GO:	0015014	heparan sulfate proteoglycan biosynthetic process, polysaccharide chain biosynthetic process	3.84E-03
GO:	0032964	collagen biosynthetic process	3.84E-03
GO:	0034244	negative regulation of transcription elongation from RNA polymerase II	3.84E-03

		promoter	
GO:	0035357	peroxisome proliferator activated receptor signaling pathway	3.84E-03
GO:	0042692	muscle cell differentiation	3.84E-03
GO:	0045792	negative regulation of cell size	3.84E-03
GO:	1900181	negative regulation of protein localization to nucleus	3.84E-03
GO:	1900748	positive regulation of vascular endothelial growth factor signaling pathway	3.84E-03
GO:	1901016	regulation of potassium ion transmembrane transporter activity	3.84E-03
GO:	0007596	blood coagulation	3.90E-03
GO:	0009888	tissue development	3.97E-03
GO:	0034329	cell junction assembly	3.97E-03
GO:	0048814	regulation of dendrite morphogenesis	3.97E-03
GO:	0010718	positive regulation of epithelial to mesenchymal transition	4.07E-03
GO:	0010975	regulation of neuron projection development	4.33E-03
GO:	0031581	hemidesmosome assembly	4.33E-03
GO:	0051895	negative regulation of focal adhesion assembly	4.33E-03
GO:	0051965	positive regulation of synapse assembly	5.10E-03
GO:	0045668	negative regulation of osteoblast differentiation	5.41E-03
GO:	1900026	positive regulation of substrate adhesion-dependent cell spreading	5.41E-03
GO:	0007346	regulation of mitotic cell cycle	5.56E-03
GO:	0001755	neural crest cell migration	5.60E-03
GO:	0038128	ERBB2 signaling pathway	5.68E-03
GO:	0060402	calcium ion transport into cytosol	5.97E-03
GO:	0090399	replicative senescence	5.97E-03
GO:	0099133	-	5.97E-03
GO:	0010628	positive regulation of gene expression	6.23E-03
GO:	0042127	regulation of cell proliferation	6.33E-03
GO:	0030010	establishment of cell polarity	6.59E-03
GO:	0030511	positive regulation of transforming growth factor beta receptor signaling pathway	6.59E-03
GO:	0010629	negative regulation of gene expression	6.63E-03

GO:	0090090	negative regulation of canonical Wnt signaling pathway	7.52E-03
GO:	0048870	cell motility	7.68E-03
GO:	0007257	activation of JUN kinase activity	7.81E-03
GO:	0050890	cognition	7.81E-03
GO:	0071364	cellular response to epidermal growth factor stimulus	7.91E-03
GO:	0043087	regulation of GTPase activity	7.94E-03
GO:	0022008	neurogenesis	7.94E-03
GO:	0032148	activation of protein kinase B activity	7.98E-03
GO:	0031573	intra-S DNA damage checkpoint	7.99E-03
GO:	0048008	platelet-derived growth factor receptor signaling pathway	7.99E-03
GO:	0051924	regulation of calcium ion transport	7.99E-03
GO:	2000249	regulation of actin cytoskeleton reorganization	7.99E-03
GO:	0001709	cell fate determination	8.12E-03
GO:	0003181	atrioventricular valve morphogenesis	8.12E-03
GO:	0006970	response to osmotic stress	8.12E-03
GO:	0030203	glycosaminoglycan metabolic process	8.12E-03
GO:	0032836	glomerular basement membrane development	8.12E-03
GO:	0040014	regulation of multicellular organism growth	8.12E-03
GO:	0043276	anoikis	8.12E-03
GO:	0050768	negative regulation of neurogenesis	8.12E-03
GO:	0050953	sensory perception of light stimulus	8.12E-03
GO:	0030334	regulation of cell migration	8.31E-03
GO:	0023014	signal transduction by protein phosphorylation	8.84E-03
GO:	0007160	cell-matrix adhesion	8.97E-03
GO:	0008037	cell recognition	9.43E-03
GO:	0042472	inner ear morphogenesis	9.43E-03
GO:	0043433	negative regulation of sequence-specific DNA binding transcription factor activity	0.01
GO:	0002576	platelet degranulation	0.01
GO:	0002315	marginal zone B cell differentiation	0.01
GO:	0031658	negative regulation of cyclin-dependent protein serine/threonine kinase activity involved in G1/S transition of mitotic cell	0.01

		cycle	
GO:	0035067	negative regulation of histone acetylation	0.01
GO:	0035108	limb morphogenesis	0.01
GO:	0035148	tube formation	0.01
GO:	0035909	aorta morphogenesis	0.01
GO:	0036289	peptidyl-serine autophosphorylation	0.01
GO:	0038202	TORC1 signaling	0.01
GO:	0048194	Golgi vesicle budding	0.01
GO:	0048384	retinoic acid receptor signaling pathway	0.01
GO:	0048646	anatomical structure formation involved in morphogenesis	0.01
GO:	2000394	positive regulation of lamellipodium morphogenesis	0.01
GO:	2001234	negative regulation of apoptotic signaling pathway	0.01
GO:	0051496	positive regulation of stress fiber assembly	0.01
GO:	0043491	protein kinase B signaling	0.01
GO:	0050731	positive regulation of peptidyl-tyrosine phosphorylation	0.01
GO:	0034383	low-density lipoprotein particle clearance	0.01
GO:	0043552	positive regulation of phosphatidylinositol 3-kinase activity	0.01
GO:	0007611	learning or memory	0.01
GO:	0006469	negative regulation of protein kinase activity	0.01
GO:	0019226	transmission of nerve impulse	0.01
GO:	0043277	apoptotic cell clearance	0.01
GO:	0048854	brain morphogenesis	0.01
GO:	0007267	cell-cell signaling	0.01
GO:	0032092	positive regulation of protein binding	0.01
GO:	0031295	T cell costimulation	0.01
GO:	0010923	negative regulation of phosphatase activity	0.01
GO:	0002159	desmosome assembly	0.01
GO:	0003197	endocardial cushion development	0.01
GO:	0007270	neuron-neuron synaptic transmission	0.01
GO:	0031915	positive regulation of synaptic plasticity	0.01
GO:	0032410	negative regulation of transporter activity	0.01
GO:	0035418	protein localization to synapse	0.01
GO:	0042976	activation of Janus kinase activity	0.01

GO:	0048842	positive regulation of axon extension involved in axon guidance	0.01
GO:	0051224	negative regulation of protein transport	0.01
GO:	0051552	flavone metabolic process	0.01
GO:	0060390	regulation of SMAD protein import into nucleus	0.01
GO:	0060575	intestinal epithelial cell differentiation	0.01
GO:	0061180	mammary gland epithelium development	0.01
GO:	0070166	enamel mineralization	0.01
GO:	0086045	membrane depolarization during AV node cell action potential	0.01
GO:	0086046	membrane depolarization during SA node cell action potential	0.01
GO:	1903243	negative regulation of cardiac muscle hypertrophy in response to stress	0.01
GO:	1903363	negative regulation of cellular protein catabolic process	0.01
GO:	0043010	camera-type eye development	0.01
GO:	0048268	clathrin coat assembly	0.01
GO:	0002265	astrocyte activation involved in immune response	0.01
GO:	0003332	negative regulation of extracellular matrix constituent secretion	0.01
GO:	0003416	endochondral bone growth	0.01
GO:	0006740	NADPH regeneration	0.01
GO:	0007617	mating behavior	0.01
GO:	0010020	chloroplast fission	0.01
GO:	0015697	quaternary ammonium group transport	0.01
GO:	0016185	synaptic vesicle budding from presynaptic endocytic zone membrane	0.01
GO:	0033292	T-tubule organization	0.01
GO:	0033600	negative regulation of mammary gland epithelial cell proliferation	0.01
GO:	0033688	regulation of osteoblast proliferation	0.01
GO:	0035408	histone H3-T6 phosphorylation	0.01
GO:	0035481	positive regulation of Notch signaling pathway involved in heart induction	0.01
GO:	0038189	neuropilin signaling pathway	0.01

GO:	0042278	purine nucleoside metabolic process	0.01
GO:	0043585	nose morphogenesis	0.01
GO:	0048671	negative regulation of collateral sprouting	0.01
GO:	0048853	forebrain morphogenesis	0.01
GO:	0051693	actin filament capping	0.01
GO:	0051705	multi-organism behavior	0.01
GO:	0060074	synapse maturation	0.01
GO:	0060219	camera-type eye photoreceptor cell differentiation	0.01
GO:	0060312	regulation of blood vessel remodeling	0.01
GO:	0070141	response to UV-A	0.01
GO:	0086048	membrane depolarization during bundle of His cell action potential	0.01
GO:	0090292	nuclear matrix anchoring at nuclear membrane	0.01
GO:	0097116	gephyrin clustering involved in postsynaptic density assembly	0.01
GO:	1900126	negative regulation of hyaluronan biosynthetic process	0.01
GO:	1902254	negative regulation of intrinsic apoptotic signaling pathway by p53 class mediator	0.01
GO:	1902339	positive regulation of apoptotic process involved in morphogenesis	0.01
GO:	1902474	positive regulation of protein localization to synapse	0.01
GO:	2000707	positive regulation of dense core granule biogenesis	0.01
GO:	2001222	regulation of neuron migration	0.01
GO:	0001889	liver development	0.01
GO:	0045104	intermediate filament cytoskeleton organization	0.01
GO:	0070509	calcium ion import	0.01
GO:	0072583	clathrin-dependent endocytosis	0.01
GO:	2000251	positive regulation of actin cytoskeleton reorganization	0.01
GO:	0007613	memory	0.02
GO:	0034446	substrate adhesion-dependent cell spreading	0.02
GO:	0050679	positive regulation of epithelial cell	0.02

		proliferation	
GO:	0043547	positive regulation of GTPase activity	0.02
GO:	0034220	ion transmembrane transport	0.02
GO:	0030324	lung development	0.02
GO:	0043066	negative regulation of apoptotic process	0.02
GO:	0035987	endodermal cell differentiation	0.02
GO:	0050919	negative chemotaxis	0.02
GO:	0006909	phagocytosis	0.02
GO:	0070372	regulation of ERK1 and ERK2 cascade	0.02
GO:	1900740	positive regulation of protein insertion into mitochondrial membrane involved in apoptotic signaling pathway	0.02
GO:	0010812	negative regulation of cell-substrate adhesion	0.02
GO:	0030878	thyroid gland development	0.02
GO:	0032801	receptor catabolic process	0.02
GO:	0048706	embryonic skeletal system development	0.02
GO:	0090286	cytoskeletal anchoring at nuclear membrane	0.02
GO:	1902287	semaphorin-plexin signaling pathway involved in axon guidance	0.02
GO:	0043647	inositol phosphate metabolic process	0.02
GO:	0010468	regulation of gene expression	0.02
GO:	0048468	cell development	0.02
GO:	0042493	response to drug	0.02
GO:	0016310	phosphorylation	0.02
GO:	0048015	phosphatidylinositol-mediated signaling	0.02
GO:	0051453	regulation of intracellular pH	0.02
GO:	0007215	glutamate receptor signaling pathway	0.02
GO:	0035723	interleukin-15-mediated signaling pathway	0.02
GO:	0045216	cell-cell junction organization	0.02
GO:	0046475	glycerophospholipid catabolic process	0.02
GO:	0050775	positive regulation of dendrite morphogenesis	0.02
GO:	0071902	positive regulation of protein serine/threonine kinase activity	0.02
GO:	0032793	positive regulation of CREB transcription factor activity	0.02
GO:	0045055	regulated exocytosis	0.02
GO:	0051899	membrane depolarization	0.02

GO:	0060349	bone morphogenesis	0.02
GO:	0048661	positive regulation of smooth muscle cell proliferation	0.02
GO:	0060412	ventricular septum morphogenesis	0.02
GO:	0033138	positive regulation of peptidyl-serine phosphorylation	0.02
GO:	0035584	calcium-mediated signaling using intracellular calcium source	0.02
GO:	0048147	negative regulation of fibroblast proliferation	0.02
GO:	0097009	energy homeostasis	0.02
GO:	0007389	pattern specification process	0.02
GO:	0030111	regulation of Wnt signaling pathway	0.02
GO:	0060291	long-term synaptic potentiation	0.02
GO:	0070555	response to interleukin-1	0.02
GO:	0001944	vasculature development	0.02
GO:	0010614	negative regulation of cardiac muscle hypertrophy	0.02
GO:	0010742	macrophage derived foam cell differentiation	0.02
GO:	0015879	carnitine transport	0.02
GO:	0032688	negative regulation of interferon-beta production	0.02
GO:	0035235	ionotropic glutamate receptor signaling pathway	0.02
GO:	0043297	apical junction assembly	0.02
GO:	0048806	genitalia development	0.02
GO:	0048846	axon extension involved in axon guidance	0.02
GO:	0051639	actin filament network formation	0.02
GO:	0070544	histone H3-K36 demethylation	0.02
GO:	0071872	cellular response to epinephrine stimulus	0.02
GO:	0086036	regulation of cardiac muscle cell membrane potential	0.02
GO:	0098943	neurotransmitter receptor transport, postsynaptic endosome to lysosome	0.02
GO:	0098970	postsynaptic neurotransmitter receptor diffusion trapping	0.02
GO:	1903800	positive regulation of production of miRNAs involved in gene silencing by miRNA	0.02
GO:	2001056	positive regulation of cysteine-type	0.02

		endopeptidase activity	
GO:	0051897	positive regulation of protein kinase B signaling	0.02
GO:	0032088	negative regulation of NF-kappaB transcription factor activity	0.02
GO:	0007043	cell-cell junction assembly	0.02
GO:	0070527	platelet aggregation	0.03
GO:	0030100	regulation of endocytosis	0.03
GO:	0035329	hippo signaling	0.03
GO:	0006357	regulation of transcription from RNA polymerase II promoter	0.03
GO:	0001946	lymphangiogenesis	0.03
GO:	0038110	interleukin-2-mediated signaling pathway	0.03
GO:	0045056	transcytosis	0.03
GO:	0050806	positive regulation of synaptic transmission	0.03
GO:	0060391	positive regulation of SMAD protein import into nucleus	0.03
GO:	0071404	cellular response to low-density lipoprotein particle stimulus	0.03
GO:	0097028	dendritic cell differentiation	0.03
GO:	0097105	presynaptic membrane assembly	0.03
GO:	0097435	fibril organization	0.03
GO:	2000573	positive regulation of DNA biosynthetic process	0.03
GO:	2001020	regulation of response to DNA damage stimulus	0.03
GO:	0030178	negative regulation of Wnt signaling pathway	0.03
GO:	0007213	G-protein coupled acetylcholine receptor signaling pathway	0.03
GO:	0010592	positive regulation of lamellipodium assembly	0.03
GO:	0010842	retina layer formation	0.03
GO:	0021766	hippocampus development	0.03
GO:	0035082	axoneme assembly	0.03
GO:	0045197	establishment or maintenance of epithelial cell apical/basal polarity	0.03
GO:	0046847	filopodium assembly	0.03
GO:	0070050	neuron cellular homeostasis	0.03

GO:	0070886	positive regulation of calcineurin-NFAT signaling cascade	0.03
GO:	0016055	Wnt signaling pathway	0.03
GO:	0060828	regulation of canonical Wnt signaling pathway	0.03
GO:	0007162	negative regulation of cell adhesion	0.03
GO:	0010971	positive regulation of G2/M transition of mitotic cell cycle	0.03
GO:	0048843	negative regulation of axon extension involved in axon guidance	0.03
GO:	0001658	branching involved in ureteric bud morphogenesis	0.03
GO:	0007221	positive regulation of transcription of Notch receptor target	0.03
GO:	0010613	positive regulation of cardiac muscle hypertrophy	0.03
GO:	0016601	Rac protein signal transduction	0.03
GO:	0035249	synaptic transmission, glutamatergic	0.03
GO:	0045742	positive regulation of epidermal growth factor receptor signaling pathway	0.03
GO:	0051402	neuron apoptotic process	0.03
GO:	0051894	positive regulation of focal adhesion assembly	0.03
GO:	0071377	cellular response to glucagon stimulus	0.03
GO:	1901379	regulation of potassium ion transmembrane transport	0.03
GO:	0007172	signal complex assembly	0.03
GO:	0007603	phototransduction, visible light	0.03
GO:	0009804	coumarin metabolic process	0.03
GO:	0018027	peptidyl-lysine dimethylation	0.03
GO:	0031290	retinal ganglion cell axon guidance	0.03
GO:	0031666	positive regulation of lipopolysaccharide-mediated signaling pathway	0.03
GO:	0032873	negative regulation of stress-activated MAPK cascade	0.03
GO:	0033160	positive regulation of protein import into nucleus, translocation	0.03

GO:	0035313	wound healing, spreading of epidermal cells	0.03
GO:	0035542	regulation of SNARE complex assembly	0.03
GO:	0035729	cellular response to hepatocyte growth factor stimulus	0.03
GO:	0035773	insulin secretion involved in cellular response to glucose stimulus	0.03
GO:	0038063	collagen-activated tyrosine kinase receptor signaling pathway	0.03
GO:	0042421	norepinephrine biosynthetic process	0.03
GO:	0042426	choline catabolic process	0.03
GO:	0045124	regulation of bone resorption	0.03
GO:	0045779	negative regulation of bone resorption	0.03
GO:	0046488	phosphatidylinositol metabolic process	0.03
GO:	0046621	negative regulation of organ growth	0.03
GO:	0050872	white fat cell differentiation	0.03
GO:	0051764	actin crosslink formation	0.03
GO:	0060339	negative regulation of type I interferon-mediated signaling pathway	0.03
GO:	0061072	iris morphogenesis	0.03
GO:	0071875	adrenergic receptor signaling pathway	0.03
GO:	0097484	dendrite extension	0.03
GO:	1903799	negative regulation of production of miRNAs involved in gene silencing by miRNA	0.03
GO:	1990869	cellular response to chemokine	0.03
GO:	0008360	regulation of cell shape	0.03
GO:	0007265	Ras protein signal transduction	0.03
GO:	0006813	potassium ion transport	0.03
GO:	0007188	adenylate cyclase-modulating G-protein coupled receptor signaling pathway	0.03
GO:	0016485	protein processing	0.03
GO:	0045766	positive regulation of angiogenesis	0.03
GO:	0043065	positive regulation of apoptotic process	0.03
GO:	0006865	amino acid transport	0.04
GO:	0032956	regulation of actin cytoskeleton organization	0.04
GO:	0030182	neuron differentiation	0.04
GO:	0000902	cell morphogenesis	0.04
GO:	0007409	axonogenesis	0.04
GO:	0007266	Rho protein signal transduction	0.04

GO:	0007040	lysosome organization	0.04
GO:	0045595	regulation of cell differentiation	0.04
GO:	0048791	calcium ion-regulated exocytosis of neurotransmitter	0.04
GO:	0006623	protein targeting to vacuole	0.04
GO:	0007016	cytoskeletal anchoring at plasma membrane	0.04
GO:	0007220	Notch receptor processing	0.04
GO:	0015695	organic cation transport	0.04
GO:	0042592	homeostatic process	0.04
GO:	0045651	positive regulation of macrophage differentiation	0.04
GO:	0045657	positive regulation of monocyte differentiation	0.04
GO:	0048557	embryonic digestive tract morphogenesis	0.04
GO:	0048699	generation of neurons	0.04
GO:	0050732	negative regulation of peptidyl-tyrosine phosphorylation	0.04
GO:	0051918	negative regulation of fibrinolysis	0.04
GO:	0071233	cellular response to leucine	0.04
GO:	0071425	hematopoietic stem cell proliferation	0.04
GO:	0071901	negative regulation of protein serine/threonine kinase activity	0.04
GO:	0090150	establishment of protein localization to membrane	0.04
GO:	0099054	presynapse assembly	0.04
GO:	0008333	endosome to lysosome transport	0.04
GO:	0070317	negative regulation of G0 to G1 transition	0.04
GO:	0086091	regulation of heart rate by cardiac conduction	0.04
GO:	0034394	protein localization to cell surface	0.04
GO:	0042177	negative regulation of protein catabolic process	0.04
GO:	0001938	positive regulation of endothelial cell proliferation	0.04
GO:	0031032	actomyosin structure organization	0.04
GO:	0050772	positive regulation of axonogenesis	0.04
GO:	0000710	meiotic mismatch repair	0.05

Supplementary Table S3. Validation of mutation through Sanger sequencing.

Sample ID#	Position	Reference sequence	Genotype
1	Chr9: 98211572	T	T/A
2	Chr9: 98211572	T	T/A
3	Chr9: 98211572	T	T/A
4	Chr9: 98211572	T	A/A
5	Chr9: 98211572	T	T/A
6	Chr9: 98211572	T	T/A

Supplementary Table S4. Univariate analysis of hazard ratio according to sites of *PTCH1* mutations in patients with breast cancer (n = 44).

	Hazard radio	95% Confidence interval	*p*-value
Exon 1	1.875	0.760-4.623	0.172
Exon 3	1.265	0.170-9.425	0.819
Exon 6	1.605	0.215-11.980	0.645
Exon 7	0.763	0.178-3.273	0.716
Exon 8	2.185	0.289-16.496	0.448
Exon 10	0.047	0-2220.028	0.578
Exon 12	0.047	0-2220.028	0.578
Exon 14	2.374	0.313-17.989	0.403
Exon 15	0.984	0.230-4.215	0.983
Exon 17	1.130	0.264-4.845	0.869
Exon 19	1.605	0.215-11.980	0.645
Exon 20	2.374	0.313-17.989	0.403
Exon 21	0.748	0.100-5.574	0.777
Exon 22	1.504	0.554-4.083	0.423
Exon 23	3.112	1.138-8.512	0.027*
Exon 22 + 23	2.496	1.063-5.861	0.036*
c.3583A>T	1.879	0.635-5.567	0.255
All sites of *PTCH1* mutation	2.491	1.062-5.843	0.036*

**p*-value less than 0.05.

Supplementary Table S5. List of PTCH1 mutation in patients with lung, liver, or distant lymph node metastasis.

Mutation site	Exon	Lung metastasis (n = 16)	Liver metastasis (n = 10)	Distant lymph node metastasis (n = 13)
c.154C>T	1	3 (19%)	3 (30%)	3 (23%)
c.158C>T	1	4 (25%)	3 (30%)	4 (31%)
c.404G>A	3	0	0	0
c.442G>A	3	0	0	0
c.466C>T	3	0	0	0
c.538G>A	3	1 (6%)	1 (10%)	1 (8%)
c.823G>A	6	1 (6%)	0	1 (8%)
c.946C>T	7	0	0	0
c.949C>T	7	0	0	0
c.957G>A	7	1 (6%)	0	1 (8%)
c.958G>A	7	0	0	0
c.973G>A	7	0	0	0
c.1018G>A	7	0	0	0
c.1180G>A	8	1 (6%)	0	1 (8%)
c.1451G>A	10	0	0	0
c.1696C>T	12	0	0	0
c.2222C>T	14	1 (6%)	0	1 (8%)
c.2251G>A	15	0	0	0
c.2335G>A	15	1 (6%)	1 (10%)	1 (8%)
c.2344C>T	15	0	0	0
c.2357G>A	15	0	0	0
c.2362T>G	15	0	0	0
c.2422C>T	15	0	0	0
c.2554C>T	15	0	0	0
c.2723G>C	17	1 (6%)	0	1 (8%)
c.2797G>A	17	1 (6%)	0	1 (8%)
c.2872G>A	17	0	0	0
c.3278G>A	19	1 (6%)	0	1 (8%)
c.3430G>A	20	1 (6%)	0	1 (8%)
c.3538C>T	21	1 (6%)	0	1 (8%)
c.3583A>T	22	4 (25%)	2 (20%)	3 (23%)
c.3610G>A	22	0	0	0
c.3641C>T	22	1 (6%)	1 (10%)	1 (8%)

c.3832C>T	23	1 (6%)	0	1 (8%)
c.3835C>T	23	1 (6%)	1 (10%)	1 (8%)
c.3844C>T	23	0	0	0
c.3907C>T	23	1 (6%)	1 (10%)	0
c.3941C>T	23	1 (6%)	1 (10%)	0
c.3992C>T	23	0	0	0

Supplementary Fig. S1. Mutation prevalence in NCKUH and TCGA. The X-axis is the total number of mutations in the particular gene. The percentage close to the bar is the number of mutations in the particular gene divided by sum of all mutations in these 15 genes.

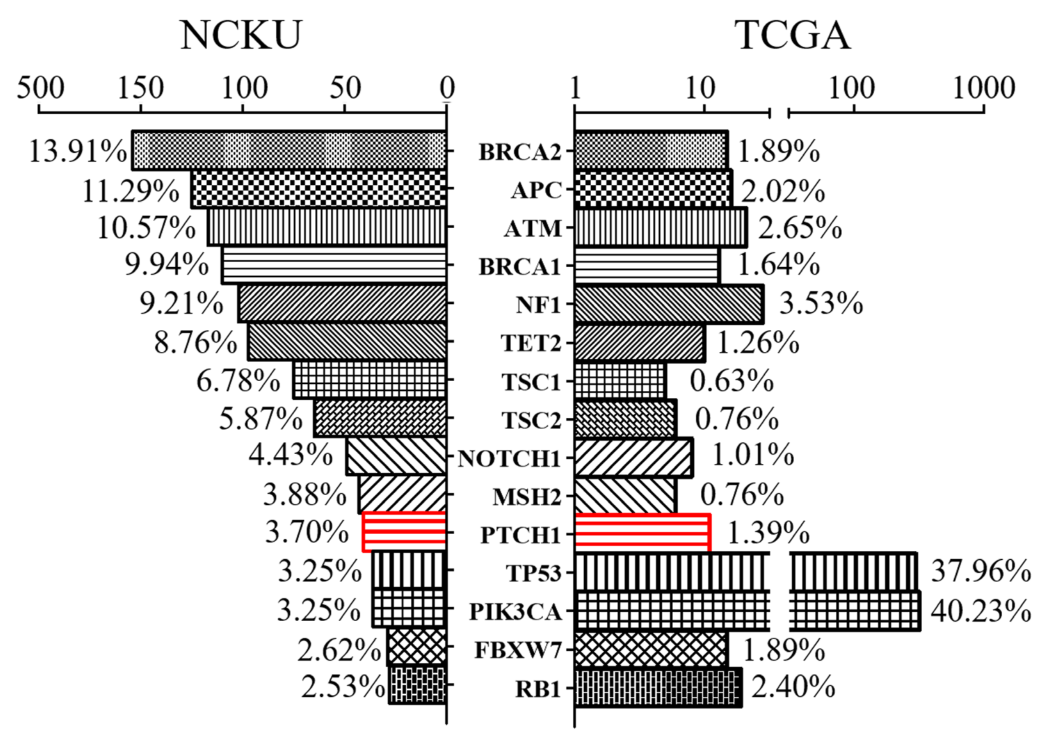

Supplementary Fig. S2. Mutational frequency of *PTCH1* gene in 44 patients with breast cancer. Total 6 patients had c.3583A>T mutation and the mutational frequency was consistently detectable.

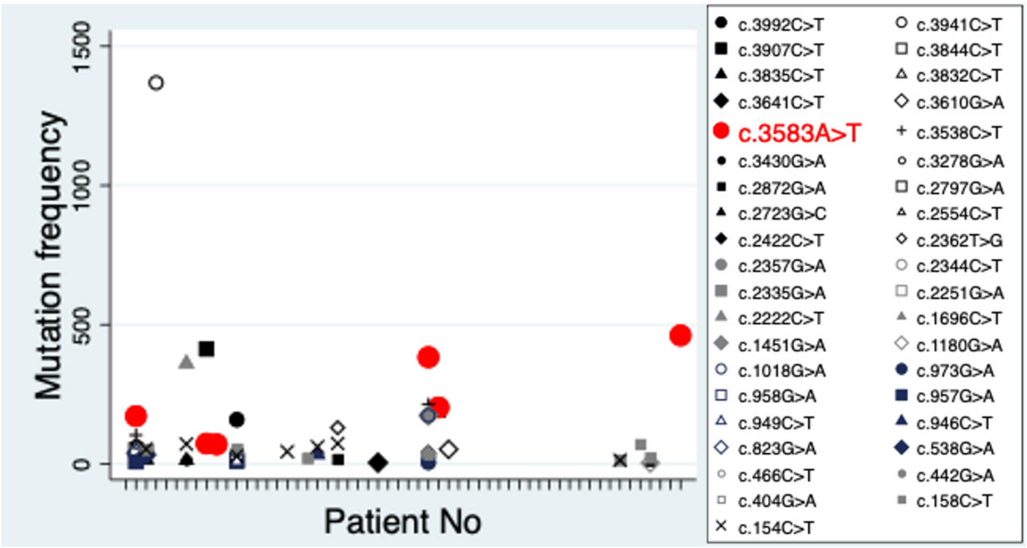

Supplementary Fig. S3. Predicted structure of wild-type *PTCH1* (left) and mutated *PTCH1* (right) by CLC genomic workbench.

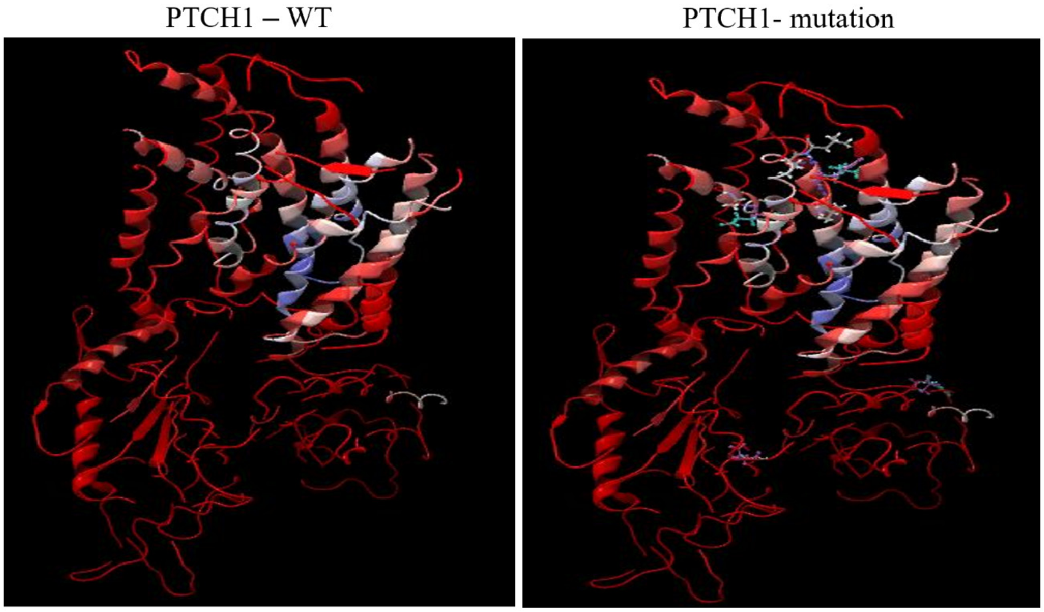

Supplementary Fig. S4. A. to **O.** Kaplan-Meier analysis of recurrence-free survival of mutations at different *PTCH1* domains according to different exon. **O.** The patients with mutated exon 23 of PTCH1 had worse survival.

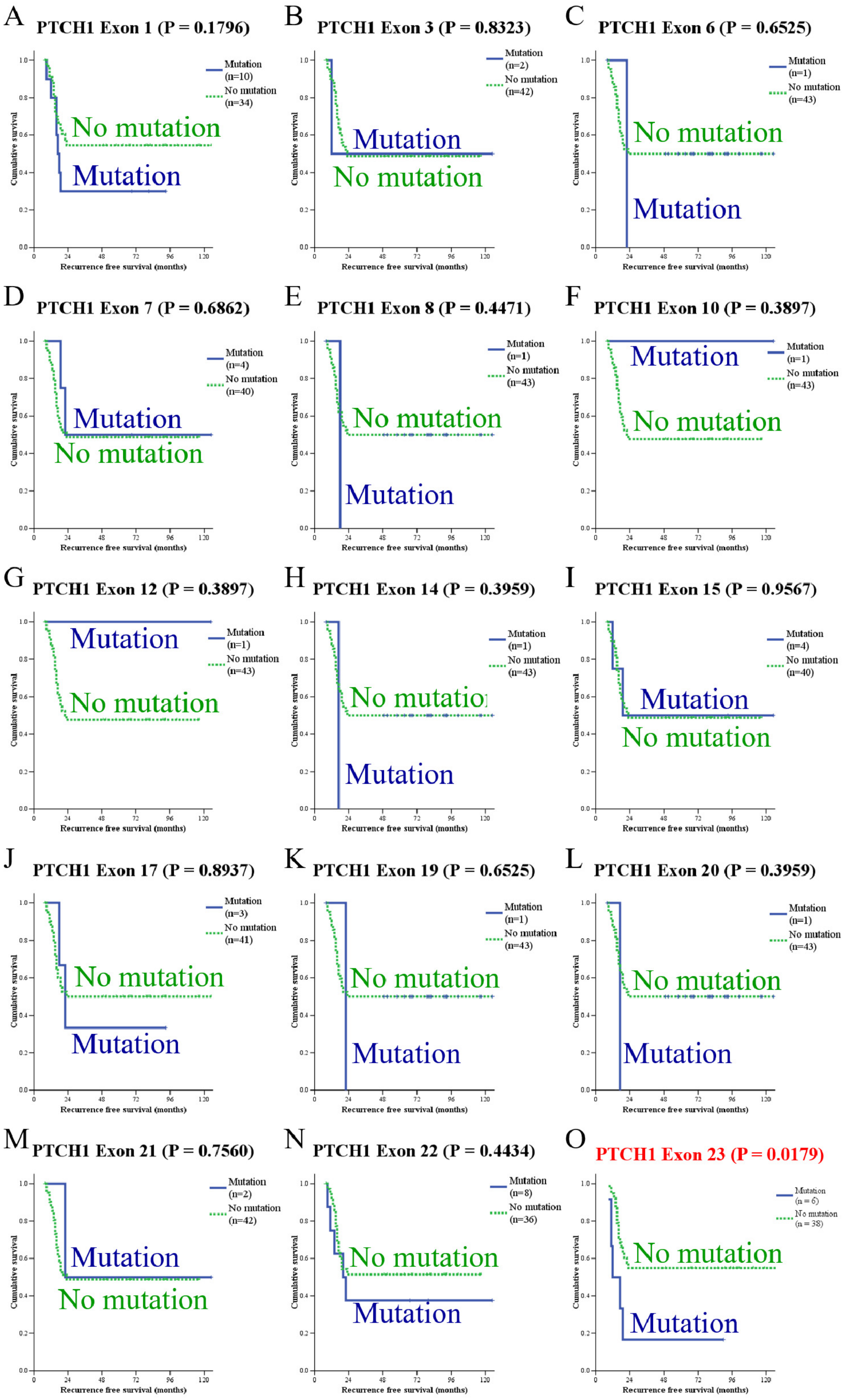

Supplement: Supplementary file 1 — Supplementary Dataset 1 [file 41598_2019_52617_MOESM1_ESM.pdf]
